# Epstein-Barr Virus-Encoded LMP1 Interacts with FGD4 to Activate Cdc42 and Thereby Promote Migration of Nasopharyngeal Carcinoma Cells

**DOI:** 10.1371/journal.ppat.1002690

**Published:** 2012-05-10

**Authors:** Hao-Ping Liu, Chia-Chun Chen, Chih-Ching Wu, Yi-Chuan Huang, Shu-Chen Liu, Ying Liang, Kai-Ping Chang, Yu-Sun Chang

**Affiliations:** 1 Molecular Medicine Research Center, Chang Gung University, Tao-Yuan, Taiwan; 2 Department of Medical Biotechnology and Laboratory Science, Chang Gung University, Tao-Yuan, Taiwan; 3 Departments of Otolaryngology-Head and Neck Surgery, Chang Gung Memorial Hospital, Lin-Kou, Taiwan; 4 Graduate Institute of Biomedical Sciences, Chang Gung University, Tao-Yuan, Taiwan; University of Wisconsin-Madison, United States of America

## Abstract

Epstein-Barr virus (EBV) is closely associated with nasopharyngeal carcinoma (NPC), a human malignancy notorious for its highly metastatic nature. Among EBV-encoded genes, latent membrane protein 1 (LMP1) is expressed in most NPC tissues and exerts oncogenicity by engaging multiple signaling pathways in a ligand-independent manner. LMP1 expression also results in actin cytoskeleton reorganization, which modulates cell morphology and cell motility— cellular process regulated by RhoGTPases, such as Cdc42. Despite the prominent association of Cdc42 activation with tumorigenesis, the molecular basis of Cdc42 activation by LMP1 in NPC cells remains to be elucidated. Here using GST-CBD (active Cdc42-binding domain) as bait in GST pull-down assays to precipitate active Cdc42 from cell lysates, we demonstrated that LMP1 acts through its transmembrane domains to preferentially induce Cdc42 activation in various types of epithelial cells, including NPC cells. Using RNA interference combined with re-introduction experiments, we identified FGD4 (FYVE, RhoGEF and PH domain containing 4) as the GEF (guanine nucleotide exchange factor) responsible for the activation of Cdc42 by LMP1. Serial deletion experiments and co-immunoprecipitation assays further revealed that ectopically expressed FGD4 modulated LMP1-mediated Cdc42 activation by interacting with LMP1. Moreover, LMP1, through its transmembrane domains, directly bound FGD4 and enhanced FGD4 activity toward Cdc42, leading to actin cytoskeleton rearrangement and increased motility of NPC cells. Depletion of FGD4 or Cdc42 significantly reduced (∼50%) the LMP1-stimulated cell motility, an effect that was partially reversed by expression of a constitutively active mutant of Cdc42. Finally, quantitative RT-PCR and immunohistochemistry analyses showed that FGD4 and LMP1 were expressed in NPC tissues, supporting the potential physiologically relevance of this mechanism in NPC. Collectively, our results not only uncover a novel mechanism underlying LMP1-mediated Cdc42 activation, namely LMP1 interaction with FGD4, but also functionally link FGD4 to NPC tumorigenesis.

## Author Summary

Epstein-Barr virus (EBV) is closely associated with human malignancies, including nasopharyngeal carcinoma (NPC). Among EBV-expressed genes, latent membrane protein 1 (LMP1) has been detected in most NPC tissues and has the ability to transform cell growth and drive cell migration, both of which are highly associated with tumorigenesis and tumor progression. Previous reports have demonstrated that cell migration primarily involves cytoskeleton rearrangement, and the RhoGTPase Cdc42 is known to actively mediate such rearrangement processes. Using LMP1-expressing NPC cells, we discovered that LMP1 induces Cdc42 activation by directly binding to FGD4, a positive regulator of Cdc42, thereby promoting motility of NPC cells. The observed correlation between FGD4 and LMP1 expression in NPC tissues provides support of physiological relevance. Notably, FGD4 has recently been shown to be responsible for a type of inherited neural disease. Our findings not only provide a novel insight into EBV pathogenesis, but also suggest a role for FGD4 in tumorigenesis.

## Introduction

Epstein–Barr virus (EBV) is a human γ-herpesvirus that is closely associated with many human malignancies, including nasopharyngeal carcinoma (NPC), Burkitt's lymphoma, T-cell lymphoma, and gastric carcinoma [1]. NPC, which is prevalent in Taiwan and southeastern China, is a human squamous cell cancer notorious for its highly metastatic nature [2]. In NPC, EBV infection is predominantly latent and viral gene expression is restricted. Among the expressed viral genes, latent membrane protein 1 (LMP1) is detected in most NPC tissues [3]. LMP1 has oncogenic properties to transform rodent fibroblast cell lines [4], [5] and promote cell growth in soft agar [6]. LMP1 is a 62-kDa integral membrane protein composed of a short N-terminal domain, six transmembrane domains, and a 200-amino-acid (aa) cytoplasmic tail at the C-terminus [7]. By mimicking TNFR (tumor necrosis factor receptor) family members, LMP1 through its cytoplasmic tail engages TRAFs (TNFR-associated factors) and TRADD (TNFR-associated death domain protein) to transduce multiple signaling pathways, including nuclear factor-kappa B (NF-κB)-mediated transcription [8] and the c-Jun amino-terminal kinase (JNK) pathway [9], [10]. Unlike TNFR-based signaling, however, LMP1 appears to signal in a ligand-independent fashion relying on its N-terminus and transmembrane domains to self-associate in the lipid rafts [11]–[13]. As a result, LMP1 is a constitutively active receptor [14]–[16].

In addition to growth transformation, LMP1 has also been linked to regulation of the actin cytoskeleton. In lymphocytes, LMP1 expression leads to the formation of membrane protrusions and membrane ruffling, which involve actin reorganization [5]. In Swiss 3T3 fibroblasts, LMP1 is capable of inducing the assembly of actin-rich surface protrusions called filopodia [17]. Moreover, the LMP1-induced formation of filopodia in fibroblasts can be abolished by a dominant-negative mutant of Cdc42, a member of the Rho (Ras-homology) GTPase family [17], implying that LMP1 is capable of activating Cdc42. Rho GTPases, mainly comprising members of the Cdc42, Rac and Rho subfamilies, actively regulate various actin-dependent functions such as cell migration, adhesion, cytokinesis, axon guidance, and phagocytosis in all eukaryotic cells [18]. Cdc42 in particular is well known to regulate actin filament (F-actin) organization and vesicle trafficking [19]–[21]. Like all GTPases, Cdc42 acts as a binary switch cycling between an inactive (GDP-bound) and an active (GTP-bound) conformational state. The activation of Cdc42 is mediated by guanine nucleotide exchange factors (GEFs), which convert the GDP-bound form of Cdc42 to the GTP-bound form [20], [22], [23]. Activated Cdc42, in turn, binds to its downstream effectors, such as the Wiskott-Aldrich syndrome protein (WASP) [24], [25], through which it ultimately generates a variety of cellular effects [26], [27]. Not surprisingly, given the prominent role of Cdc42 in so many aspects of cell biology, aberrant activation of Cdc42 (or dysfunction of Cdc42 GEFs) results in pathogenesis, including tumorigenesis and tumor progression, cardiovascular diseases, diabetes, and neuronal degenerative diseases [28], [29].

FGD4 (FYVE, RhoGEF and PH domain-containing 4), also known as Frabin (FGD1-related F-actin binding protein), like FGD2 and FGD3, is a Cdc42-specific GEF that shows significant sequence homology to FGD1, which was originally discovered by positional cloning as the gene responsible for a human X-linked skeletal disease called faciogenital dysplasia [30]–[33]. It has been revealed that mutations in the gene encoding FGD4 cause an inherited neurological disease commonly referred to as Charcot-Marie-Tooth (CMT) disease, a type of hereditary motor and sensory neuropathy [34]. All FGD proteins possess a similar domain organization, whereas each FGD has a unique N-terminal region [35]. FGD4 consists of an N-terminal FAB (F-actin-binding) domain, a DH (Dbl homology) domain containing the principal GEF catalytic unit, and multiple phosphoinositide-binding domains, including two PH (pleckstrin homology) domains and an FYVE (Fab1, YOTB, Vac1, and EEA1) domain at the C-terminus [30], [35]. Accordingly, FGD4 likely couples the actin cytoskeleton to the cellular membrane by localization to the membrane and simultaneously binding F-actin. In fibroblasts, it has been shown that rat Fgd4 binds along the sides of F-actin through the FAB domain and directly induces activation of Cdc42 in the vicinity of actin structures, resulting in actin reorganization [30]. However, the mechanisms by which external or internal stimuli transduce the signals to activate FGD4 largely remain unclear.

In the present study, we sought to investigate the activation of Cdc42 by LMP1 in NPC cells, which are physiologically relevant to EBV. Importantly, we uncovered a novel mechanism underlying LMP1-mediated Cdc42 activation, showing that LMP1 physically interacts with FGD4, leading to functional consequences associated with NPC tumorigenesis and tumor progression.

## Results

### LMP1 preferentially induces Cdc42 activation in epithelial cells

**Figure 1:**
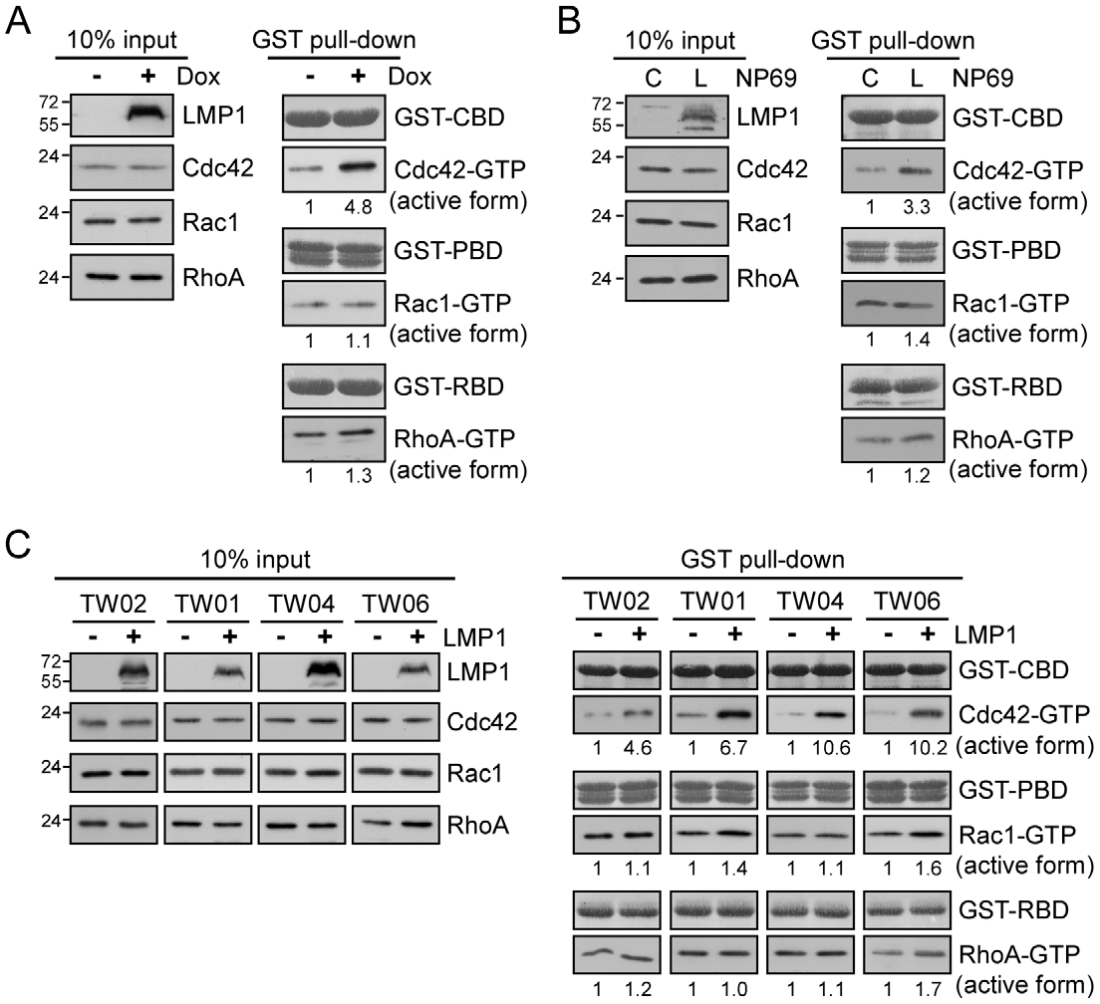
LMP1 preferentially induces Cdc42 activation in epithelial cells. (A) 293 Tet-On cells were treated with 5 µg/ml doxycyclin (Dox) for 24 h to induce LMP1 expression. After a 6-h serum starvation, cells were lysed and the lysates were incubated with immobilized GST-CBD (active Cdc42-binding domain of WASP), GST-PBD (active Rac1-binding domain of PAK1), or GST-RBD (active RhoA-binding domain of Rhoteckin) at 4°C for 1 h to pull down the active form of Cdc42, Rac1, or RhoA in cells. Details are provided in Materials and Methods. Total (input) and active (GTP-bound) levels of Cdc42, Rac1, or RhoA were analyzed by Western blotting with the respective antibody. Numbers represent relative fold-changes of GTPase activation determined based on densitometric quantitation. The trace in the absence of LMP1 was set as 1. The constant amounts of GST-fusion proteins for pull-down assays were shown by Ponceau S staining. (B) Cell lysates from NP69 cells (denoted C) or stable LMP1-expressing NP69 cells (denoted L) incubated in serum-free media for 6 h were harvested for GST pull-down assays, and levels of active Cdc42, Rac1, and RhoA were assessed as described above. (C) A variety of NPC cell lines, NPC-TW01, -TW02, -TW04, and -TW06, were transfected with an LMP1 expression plasmid or empty vector (control) and incubated for 24 h. Following a 6-h serum starvation, cells were lysed and the lysates were applied to GST pull-down assays. The resulting products were analyzed for active Cdc42, Rac1, and RhoA, as described above.

To assess the effect of LMP1 on Cdc42 activation in cells, we carried out GST-pull-down assays using GST-CBD (containing the active Cdc42-binding domain of WASP) as bait to precipitate active Cdc42 in lysates of 293 Tet-On cells, in which the expression of LMP1 was induced by doxycycline (Dox). As shown in Figure 1A, LMP1 expression led to a 4.8-fold increase in the level of active Cdc42 compared with the control without affecting total Cdc42 expression levels. Moreover, LMP1-mediated Cdc42 activation was specific since activation of Rac1 and RhoA, two related members of Rho GTPase family, was not significant.

To corroborate these phenomena in EBV-associated cells, we conducted similar GST pull-down assays using nasopharyngeal epithelial cells (NP69) and four NPC cell lines, each of which expressed LMP1 or empty vector (control). Consistent with the results obtained in 293 Tet-On cells, LMP1 expression led to a 3.3-fold increase in active Cdc42 in NP69 cells (Figure 1B), and induced 4.6-, 6.7-, 10.2-, and 10.6-fold increases in active Cdc42 in four tested NPC cell lines (TW02, TW01, TW04, and TW06), respectively (Figure 1C). In contrast, there was no evidence for Rac1 or RhoA activation by LMP1 in NP69 cells or NPC cells (Figure 1B and 1C). Collectively, these results demonstrate that LMP1 preferentially induces Cdc42 activation in various types of epithelial cells.

### LMP1 transmembrane domains are required for Cdc42 activation

**Figure 2:**
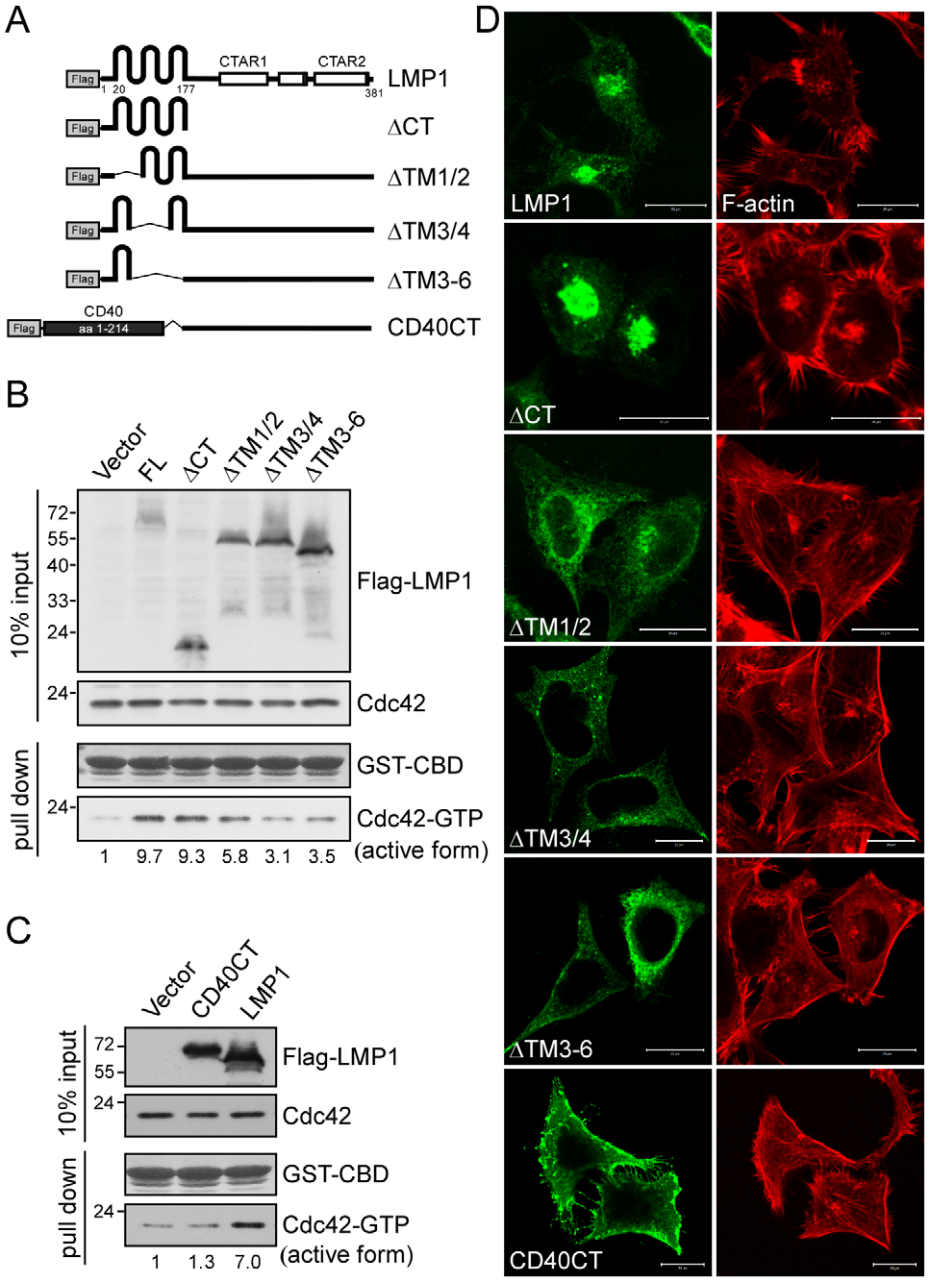
The transmembrane domains of LMP1 are required for induction of Cdc42 activation and the associated F-actin rearrangement. (A) Schematic illustrations of Flag-tagged LMP1 and its truncated derivatives. FL, full-length LMP1; ΔCT, deletion of the C-terminus; ΔTM1/2, deletion of transmembrane domains 1 and 2; ΔTM3/4, deletion of transmembrane domains 3 and 4; ΔTM3–6, deletion of transmembrane domains 3–6. CD40CT indicates that the entire transmembrane region of LMP1 (aa 1–177) is replaced with that of CD40 (aa 1–214). (B) NPC-TW04 cells that had been transfected with 1 µg of plasmid encoding Flag-LMP1 or its truncated versions were grown for 24 h, followed by serum starvation for 6 h. Then cell lysates were harvested for GST pull-down assays using GST-CBD beads as bait (detailed in Materials and Methods) to pull-down active (GTP-bound) Cdc42 in cells. The resulting products were analyzed by Western blotting with an anti-Cdc42 antibody. The levels of active Cdc42 were quantified as described above. The trace in the absence of LMP1 was set as 1. The constant amounts of GST-CBD used in pull-down assays were shown by Ponceau S staining. (C) NPC-TW04 cells that had been transfected with 1 µg of plasmid encoding Flag-LMP1 or the chimera CD40CT were lysed after incubation as described above. Cell lysates were harvested for GST-CBD pull-down assays, and the level of active Cdc42 was determined as described above. (D) Induction of F-actin rearrangement by LMP1. NPC-TW04 cells that had been grown on poly-L-lysine-coated coverslips overnight were transfected with 0.5 µg of plasmid encoding Flag-tagged full-length, truncated, or chimeric LMP1, and then incubated for 24 h. Following a 6-h serum starvation, cells were fixed and permeabilized, followed by subsequent staining with primary anti-Flag (M2) and FITC-conjugated secondary antibodies. For actin filament (F-actin) staining, cells were co-stained with TRITC-conjugated phalloidin. Images were acquired using a ZEISS LSM510 confocal microscope as detailed in Materials and Methods. Scale bars, 20 µm. Transfection of an empty vector had no effect on actin organization (data not shown).

To dissect the functional domains of LMP1 responsible for its activation of Cdc42, we generated a series of LMP1 deletion constructs (Figure 2A) and examined their effects on Cdc42 activation in NPC cells. Deletion mutants lacking transmembrane domains 3 and 4 (ΔTM3/4) or 3–6 (ΔTM3–6) exhibited substantially impaired ability to activate Cdc42, producing 3.1- and 3.5-fold increases, respectively, compared to the 9.7-fold increase induced by full-length LMP1 (Figure 2B). In contrast, deletion of the entire C-terminal cytoplasmic tail (ΔCT) of LMP1 did not interfere with Cdc42 activation (a 9.3-fold vs. a 9.7-fold increase), indicating that C-terminus-dependent LMP1-transduced signaling pathways are not involved in this event. To confirm the importance of the transmembrane domains of LMP1 in Cdc42 activation, we replaced this region with the transmembrane domain of a TNFR member, CD40 (denoted CD40CT in Figure 2A). As shown in Figure 2C, the resulting chimera failed to activate Cdc42 (1.3-fold increase vs. 7.0-fold increase for chimeric and wild-type LMP1, respectively), indicating that the transmembrane domains of LMP1 are required for Cdc42 activation. To verify whether the LMP1-induced activation of Cdc42 was associated with remodeling of the actin filaments, we next conducted the immunofluorescence staining using NPC cells which expressed Flag-LMP1 or its various transmembrane domains-truncated forms. As shown in Figure 2D, expression of Flag-LMP1 and its C-terminus-deleted form (ΔCT) led to formation of microspike-like actin structures (filopodia) at the plasma membrane and actin bundles at the perinuclear regions (Golgi apparatus). In contrast, expression of the transmembrane domains-truncated form ΔTM3/4 or ΔTM3–6, or the chimera CD40CT failed to induce the actin remodeling as described above. The truncated form ΔTM1/2 remained a moderate ability for the actin remodeling compared to the ΔTM3–6 or the CD40CT chimera, correlating with the extent of Cdc42 activation.

Since the transmembrane domains confer properties on LMP1 that distinguish it from TNFR family members [13], [14], we next investigated whether the TNFR members, TNFR and IL-1R (interleukin-1 receptor), were capable of activating Cdc42 in NPC cells. Compared with vehicle controls, treatment of NPC cells with the TNFR ligand TNF-α (50 or 100 ng/ml) or the IL-1R ligand IL-1α (10 ng/ml) for 30 min had little effect on Cdc42 activation (Figure S1A) and on actin remodeling (Figure S1B). While NPC cells seemed to be insensitive to IL-1α induction of NF-κB signaling, positive controls showed that TNF-α induced degradation of inhibitor kappa B (IκBα) protein (Figure S1A) and subsequent nuclear translocation of a NF-κB subunit p65 (Figure S1B), indicating activation of NF-κB signaling [36] and confirming the functional integrity of TNF-α in this experimental setting. These data conclusively demonstrated that the action of LMP1 was distinct from that of TNFR with respect to Cdc42 activation in NPC cells.

### LMP1 appears to induce Cdc42 activation at LMP1-resident sites

To better elucidate the nature of LMP1-mediated Cdc42 activation, we sought to dissect the spatial pattern of Cdc42 activation upon LMP1 expression. For this aim, we generated an EGFP-CBD construct containing the active Cdc42-binding domain of WASP to locate active Cdc42 inside cells. Following transfection of 293 Tet-On cells with pEGFP-CBD, LMP1 expression was induced by doxycycline (Dox) and cells were analyzed by confocal microscopy. As shown in Figure S2A (upper panel), LMP1 expression led to EGFP-CBD co-distribution at LMP1-resident sites (i.e., Golgi apparatus and plasma membrane). In contrast, a more homogenous distribution of EGFP-CBD was evident in cells in which LMP1 expression was not induced (bottom panel), implying Cdc42 activation at LMP1-resident sites. We next verified whether LMP1 expression resulted in distribution of Cdc42 to LMP1-resident sites using NPC cells expressing EGFP-LMP1 or the truncated form ΔTM3–6. As shown in Figure S2B, a portion of Cdc42 was co-distributed with EGFP-LMP1 (the inset) rather than with the ΔTM3–6 form in NPC cells. These data suggest the possibility that certain factors that act upstream of Cdc42-activation cascades may co-localize with LMP1 and participate in this event.

### FGD4 is the GEF responsible for LMP1-mediated Cdc42 activation

**Figure 3:**
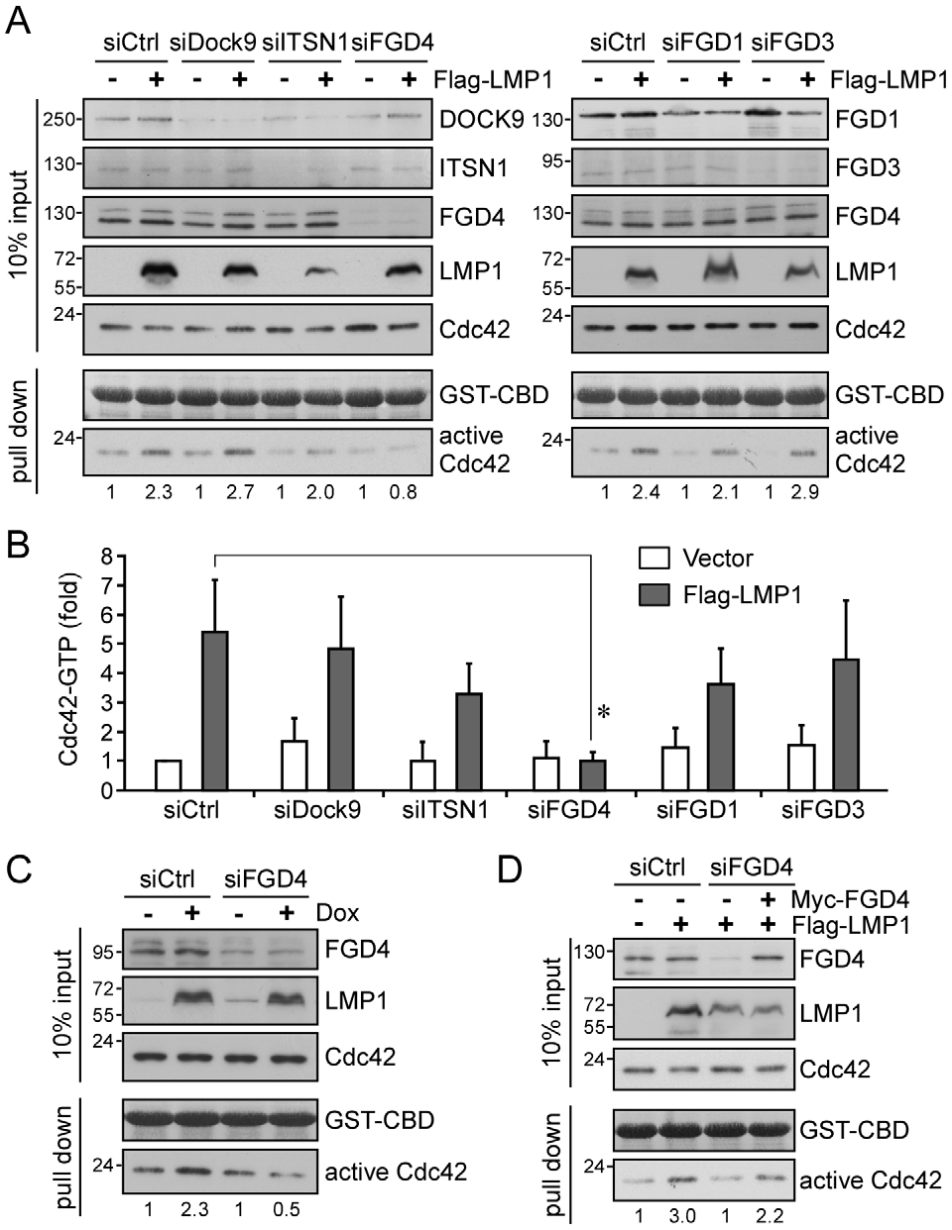
FGD4, a Cdc42 GEF, is involved in LMP1-mediated Cdc42 activation. (A) Identification of FGD4 as a GEF candidate involved in LMP1-mediated Cdc42 activation in NPC cells. The potential GEF involved in the activation of Cdc42 by LMP1 was identified by co-transfecting NPC-TW04 cells with 1 µg of Flag-LMP1 expression plasmid or empty vector (control), and siRNAs (25 µM) directed against DOCK9 (siDock9), intersectin-1 (siITSN1), FGD4 (siFGD4), FGD1 (siFGD1), FGD3 (siFGD3) or non-targeting control (siCtrl). Following a 6-h serum starvation at 48 h post-transfection, cells lysates were harvested for GST-CBD pull-down assays. The knockdown efficiency for individual GEFs was analyzed by Western blotting with the respective antibody. The level of active Cdc42 was determined as described above. The constant amounts of GST-CBD for pull-down assays were shown by Ponceau S staining. The results shown are representative of five independent experiments. Quantitative data are shown in B. The relative fold-changes in active Cdc42 are presented as means ± SDs (*P = 0.0052; paired *t*-test). (C) Confirmation of FGD4 involvement in 293 Tet-On cells. 293 Tet-On cells that had been transfected with 25 µM control or FGD4 siRNA and incubated for 24 h were further treated with Dox (5 µg/ml) for 24 h to induce LMP1 expression. Cell extracts were then harvested for GST-CBD pull-down assays and the level of active Cdc42 was determined as described above. (D) Normalizing the reduced levels of active Cdc42 in FGD4-depleted cells by re-introduction of FGD4. NPC-TW04 cells were co-transfected with 25 µM control or FGD4 siRNA, together with 1 µg of Flag-LMP1 expression plasmid or empty vector (control), plus 2 µg of pMyc-FGD4 expression plasmids or Myc vector. Following a 6-h serum starvation at 48 h post-transfection, cells lysates were harvested for GST-CBD pull-down assays, and the level of active Cdc42 was analyzed as described above.

On the basis of the above observation (Figures 2 and S2) and the preferential activation of Cdc42 by LMP1 (Figure 1), we hypothesized that LMP1 modulates a Cdc42 GEF and thereby activates Cdc42. To verify this, we first surveyed the literature for candidate Rho GEFs that show specificity toward Cdc42 and localize to the Golgi apparatus and the plasma membrane, where LMP1 was expressed as well (Figure 2E). A subset of GEFs that meet such criteria, including FGD1 [37], FGD3 [32], FGD4 [38], intersectin-1 (ITSN1) [39], and DOCK9 (zizimin) [40], was selected for further evaluation. The expression of these GEFs at the transcriptional level was validated in NPC cells using quantitative reverse transcription-polymerase chain reaction (RT-PCR; data not shown). To evaluate the effects of these GEFs on LMP1-mediated Cdc42 activation, we performed RNA interference using small interfering RNA (siRNA) to deplete each GEF from LMP1-expressing NPC cells, followed by precipitation of active Cdc42, as described above. The knockdown efficiency of each siRNA toward its targeted GEF was analyzed by quantitative RT-PCR (Figure S3A) and Western blotting. As shown in Figure 3A, knockdown of FGD4 (siFGD4) reduced the LMP1-induced Cdc42 activation from the 2.3-fold increase observed in control siRNA (siCtrl)-treated cells to a 0.8-fold increase. In contrast, knockdown of each of the other GEFs had little effect on LMP1-induced Cdc42 activation. A quantitative analysis of data from five independent experiments further reinforced the inhibitory effect of FGD4 knockdown on LMP1-induced Cdc42 activation (Figure 3B). To reproduce the phenomenon in epithelial cells other than NPC cells, we carried out similar knockdown experiments using 293 Tet-On cells with or without Dox induction. As shown in Figure 3C, knockdown of FGD4 consistently reversed LMP1-mediated Cdc42 activation, reducing the fold-increase from 2.3 to 0.5, indicating that this effect was not restricted to NPC cells.

To reinforce the functional role of FGD4, we re-introduced FGD4 expression by ectopically expressing FGD4 (Myc-FGD4) in FGD4-depleted NPC cells that co-expressed LMP1, and then assessed the activation of Cdc42 by LMP1. As shown in Figure 3D, depletion of FGD4 consistently eliminated LMP1-mediated Cdc42 activation, reducing Cdc42 activation from a 3.0-fold increase to a 1.0-fold increase. In contrast, re-introduction of FGD4 expression attenuated this reduction, limiting it to a 2.2-fold increase. Collectively, these results confirm that FGD4 indeed plays a role in the activation of Cdc42 by LMP1. As noted, FGD4 depletion had no effect on the basal level of Cdc42 activation in cells lacking LMP1 (Figure 3). Accordingly, the data demonstrate that FGD4 is primarily involved in mediating LMP1-induced Cdc42 activation.

### FGD4 modulates LMP1-induced Cdc42 activation through protein-protein interaction

**Figure 4:**
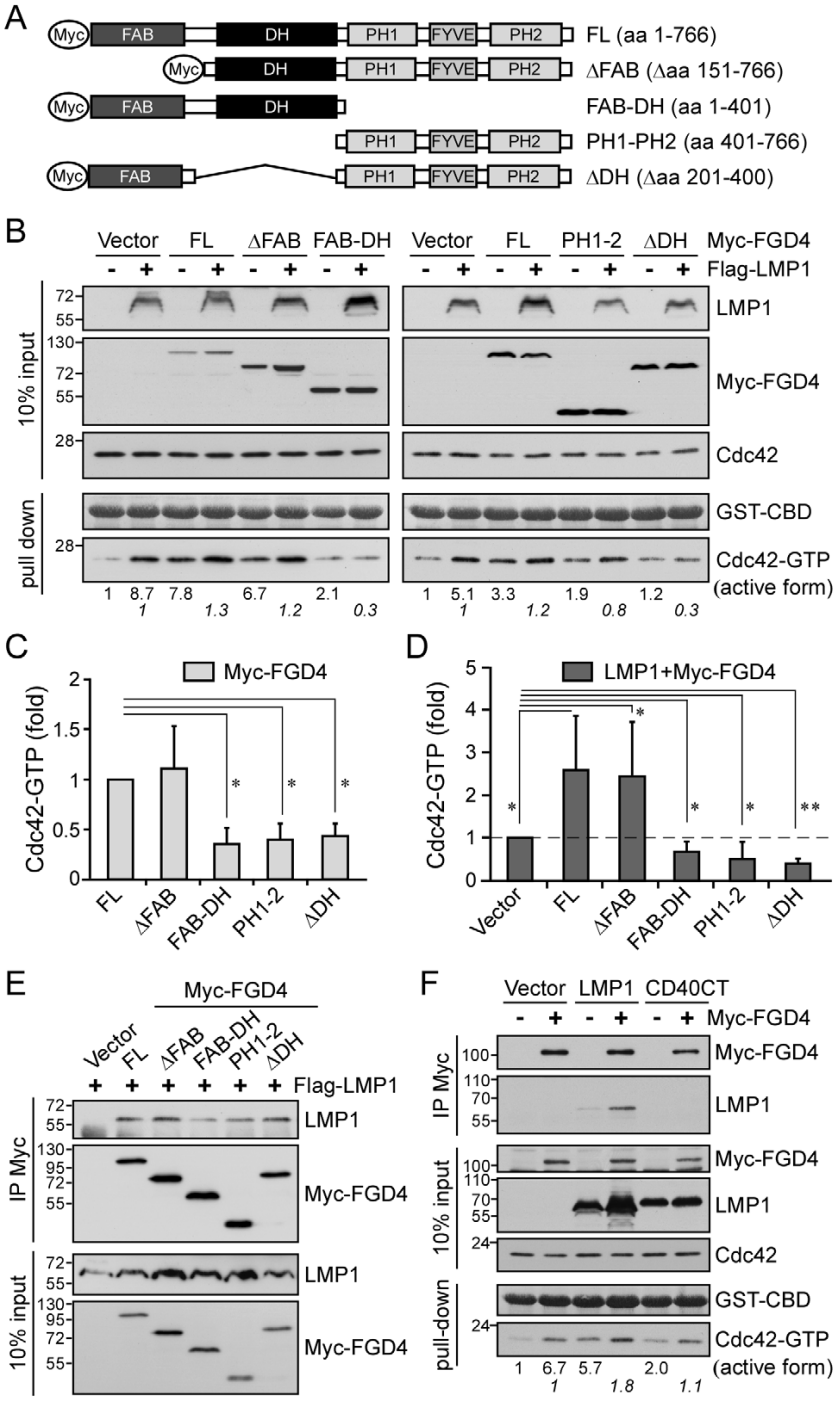
FGD4 modulates LMP1-mediated Cdc42 activation through protein-protein interaction. (A) Schematic illustrations of Myc-tagged FGD4 and its truncated derivatives. FL, full-length FGD4; ΔFAB, deletion of the F-actin-binding domain; ΔDH, deletion of the Dbl homology domain (catalytic domain); PH1 and PH2, pleckstrin homology domains 1 and 2; FYVE, a zinc finger domain named after Fab1, YOTB, Vac1, and EEA1. (B) Characterization of FGD4 function and its effect on LMP1-mediated Cdc42 activation. NPC-TW01 cells were co-transfected with 1.5 µg of Myc-FGD4 expression plasmids and 1 µg of Flag-LMP1 expression plasmid or empty vector (control). After 24-h incubation and a following 6-h serum starvation, cells were lysed for GST-CBD pull-down assays and the level of active Cdc42 was determined as described above. The constant amounts of GST-CBD for pull-down assays were shown by Ponceau S staining. The domain of FGD4 required for Cdc42 activation was determined by calculating the relative fold-change in active Cdc42 with expression of truncated FGD4 versus expression of full-length FGD4. The mean ± SD of five independent experiments is shown in C (*, *P*<0.001; paired *t*-test). The effect of FGD4 on LMP1-mediated Cdc42 activation was demonstrated by calculating the relative fold-change in active Cdc42 with expression of both LMP1 and FGD4 versus expression of LMP1 alone (B; numbers in italics). The mean ± SD of five independent experiments is shown in D (*P<0.05, **P<0.01; paired *t*-test). (E) Co-immunoprecipitation of Flag-LMP1 with Myc-FGD4. NPC-TW01 cells that had been transfected with the indicated plasmids were lysed after incubation as described in B. The resulting cell lysates were applied to co-immunoprecipitation assays with an anti-Myc affinity matrix. The precipitated protein complexes were analyzed by Western blotting with anti-LMP1 (S12) and anti-Myc (9E10) antibodies. (F) NPC-TW01 cells that had been transfected with plasmids encoding Flag-LMP1 or Flag-CD40CT together with Myc-FGD4 were lysed after incubation as described in B. A portion of the resulting cell lysate was analyzed by co-immunoprecipitation assays using an ani-Myc affinity matrix and the remainder was analyzed by GST-CBD pull-down assays, as described above.

What little has been learned to date about the function of FGD4 has mainly been gleaned from experiments involving manipulation of rat Fgd4 [30], [35], [38], [41]. Despite the high sequence homology between rat Fgd4 and human FGD4 (Figure S3B), it is uncertain whether the functions of both proteins are identical. To characterize the function of human FGD4 in NPC cells, we generated a series of truncated human FGD4 constructs (Figure 4A) to examine their effects on Cdc42 activation (Figure 4B–4D) and actin remodeling (Figure S4A). As shown in Figure 4B, expression of Myc-FGD4 (FL) activated Cdc42 compared with the vector control, increasing the level of active Cdc42 by 3.3- (right panel) and 7.8-fold (left panel) in two different experiments. The immunofluorescence staining revealed that expression of Myc-FGD4 induced filopodia formation at the plasma membrane (Figure S4A). Deletion of the FAB domain (ΔFAB) of FGD4 did not affect the activation of Cdc42 (Figure 4C) and the induction of filopodia (Figure S4A). In contrast, deletion of the DH domain (ΔDH vs. FL; PH1–2 vs. ΔFAB) substantially impaired the activity of FGD4 toward Cdc42 (Figure 4B and 4C) as well as induction of actin remodeling (Figure S4A) and recruitment of Cdc42 (Figure S4B), indicating that the DH domain is essential for a full FGD4 activity. Moreover, deletion of the PH1-to-PH2 domains (denoted FAB–DH) caused a functional impairment of FGD4 (Figures 4B, 4C, and S4A) but left its ability to recruit Cdc42 unchanged (Figure S4B), suggesting some aspect of the function of PH1-to-PH2 domains, such as membrane targeting, is also needed for a full FGD4 activity.

To investigate how FGD4 affects LMP1-mediated Cdc42 activation, we assessed the activation of Cdc42 by LMP1 in NPC cells expressing LMP1 together with various forms of FGD4. As shown in Figure 4B, ex`pression of LMP1 alone led to activation of Cdc42 compared with vector-transfected cells, increasing active Cdc42 levels by 5.1- (right panel) and 8.7-fold (left panel) in two different experiments. Co-expression of LMP1 with the full-length FGD4 and with the ΔFAB form further augmented LMP1-mediated Cdc42 activation by 1.2- and 1.3-fold, respectively (Figure 4B), in agreement with the intact activities of these FGD4 proteins (Figure 4C). In contrast, co-expression of LMP1 with the truncated forms of FGD4 that exhibited impaired FGD4 activities (ΔDH, PH1–2, and FAB–DH) instead reduced LMP1-mediated Cdc42 activation on average by 62%, 50%, and 39%, respectively (Figure 4D), revealing that FGD4 indeed acts downstream of LMP1 to modulate Cdc42 activation.

On the basis of the known ability of PH domains to bind to phosphoinositides as well as proteins [42], [43] and our observation that LMP1-mediated Cdc42 activation occurred at LMP1-resident sites (Figure S2), we speculated that LMP1 likely interacted with FGD4. To verify the interaction between LMP1 and FGD4, we performed co-immunoprecipitation assays using NPC cells co-expressing LMP1 and various forms of FGD4. As shown in Figure 4E, LMP1 was co-precipitated with full-length, ΔFAB, and ΔDH forms of FGD4, indicating that LMP1 interacted with FGD4 in a manner that did not require FAB or DH domains. Consistent with this, the FAB–DH form showed an impaired ability to interact with LMP1, in contrast to the PH1–2 form, which was sufficient for interaction with LMP1. Collectively, these results revealed that the PH1-to-PH2 domains of FGD4 are mainly responsible for the interaction of FGD4 with LMP1.

### LMP1 transmembrane domains are required for the interaction with FGD4 and the promotion of FGD4 activity

To corroborate the impact of the LMP1-FGD4 interaction on LMP1 activation of Cdc42, we next examined if the transmembrane domains of LMP1, which were necessary for inducing Cdc42 activation (Figure 2B and 2C), were responsible for the interaction with FGD4. To accomplish this, we performed co-immunoprecipitation assays using anti-Myc affinity resins to precipitate Myc-FGD4-associated protein complexes in lysates of NPC cells co-expressing Myc-FGD4 plus Flag-LMP1 or its chimera, CD40CT. As shown in Figure 4F, wild-type LMP1 but not CD40CT was co-precipitated with Myc-FGD4, indicating that the transmembrane domains of LMP1 are required for its interaction with FGD4. To explore the role of this protein-protein interaction in FGD4 activity, we assessed Cdc42 activation by Myc-FGD4 in the presence of LMP1 or CD40CT. The results showed that co-expression of Myc-FGD4 with LMP1 increased the level of active Cdc42 by 1.8-fold compared with expression of FGD4 alone (Figure 4F). In contrast, co-expression of FGD4 with CD40CT did not promote Cdc42 activation (1.1-fold). Taken together, these data revealed that LMP1 interacts with and coordinates the activity of FGD4.

**Figure 5:**
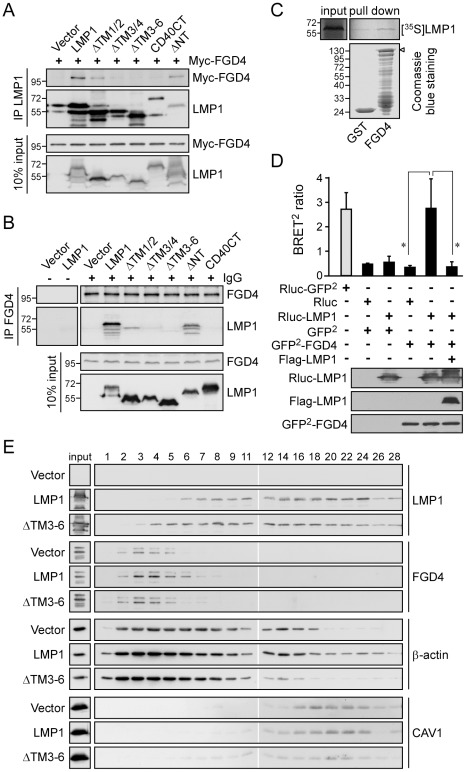
LMP1 via its transmembrane domains 3 and 4 interacts with FGD4 and leads to redistribution of actin. (A) Co-immunoprecipitation of Myc-FGD4 with Flag-LMP1. NPC-TW04 cells were co-transfected with 2 µg of expression plasmids for Myc-FGD4 plus 1.5 µg of expression plasmids for Flag-tagged full-length, truncated, or chimeric LMP1. Cells were lysed at 24 h post-transfection, and the resulting cell lysates were analyzed by co-immunoprecipitation assays using an anti-Flag affinity matrix. Details are provided in Materials and Methods. After an overnight incubation at 4°C, the precipitated protein complexes were analyzed by Western blotting using anti-Myc and anti-Flag antibodies. (B) Co-immunoprecipitation of Flag-LMP1 with endogenous FGD4. NPC-TW04 cells transfected with 2 µg of expression plasmids for Flag-LMP1, its truncated forms, or CD40CT chimera were lysed at 24 h post-transfection. The resulting cell lysates were analyzed by co-immunoprecipitation assays using anti-FGD4 antibodies coupled with protein G beads. Details are provided in Materials and Methods. After an overnight incubation at 4°C, the precipitated protein complexes were analyzed by Western blotting using anti-FGD4 and anti-LMP1 antibodies. (C) *In vitro* binding of LMP1 to FGD4. Equal amounts of *in vitro*-translated, [^35^S]methionine-labeled LMP1 proteins were incubated with immobilized GST or GST-FGD4, as detailed in Materials and Methods. The bound LMP1 proteins were resolved by SDS-PAGE followed by autoradiography. The amounts of GST-fusion proteins (arrowhead) were determined by Coomassie blue staining. (D) Detection of a direct interaction between LMP1 and FGD4 in living cells using BRET^2^ assays. BRET^2^ ratios were measured in NPC-TW04 cells expressing the indicated constructs. Cells were detached at 24 h post-transfection and analyzed by BRET^2^ assays as described in Materials and Methods. Expression of the GFP^2^-fused *Renilla* luciferase (Rluc-GFP^2^) served as a positive control. The data represent the means ± SDs of four independent readings from one representative result of four independent experiments (*P<0.01; paired *t*-test). Expression levels of the indicated fusion proteins were determined by Western blot analysis of a portion of cell lysates using the respective antibodies. (E) NPC-TW04 cells were transfected with 1 µg of plasmid for Flag-LMP1, its truncated form (ΔTM3–6), or empty vector, and then grown for 24 h. Cell lysates were individually harvested for subcellular fractionation as described in Materials and Methods. Every fraction from fractions 1–9 and every other fraction from fractions 11–28 were analyzed by Western blotting with specific antibodies against LMP1, FGD4, β-actin, and CAV1.

To investigate whether Myc-FGD4 was co-precipitated with LMP1 in a reciprocal way, we conducted co-immunoprecipitation assays using anti-Flag affinity resins to precipitate Flag-LMP1-associated protein complexes in lysates of NPC cells co-expressing Myc-FGD4 with various forms of LMP1. As shown in Figure 5A, Myc-FGD4 was co-precipitated with Flag-LMP1 but not with the CD40CT chimera, consistent with the result shown in Figure 4F. In contrast, Myc-FGD4 was not co-precipitated with the truncated form ΔTM3/4 or ΔTM3–6, indicating that the transmembrane domains 3 and 4 were the minimal region required for LMP1 interaction with FGD4. Moreover, deletion of the short N-terminal domain (ΔNT) did not affect LMP1 co-precipitation of Myc-FGD4 (Figure 5A). We next corroborate the interaction between endogenous FGD4 and LMP1 by co-immunoprecipitation assays using NPC cells expressing various forms of LMP1. The resulting cell lysates were subsequently incubated with an anti-FGD4 antibody coupled with protein G beads to precipitate endogenous FGD4. As shown in Figure 5B, Flag-LMP1 was co-precipitated with FGD4, indicating a physical interaction between FGD4 and LMP1. Notably, neither the CD40CT chimera nor the LMP1 truncated form lacking the transmembrane domains 3 and 4 (ΔTM3/4 and ΔTM3–6) could be detected in the FGD4-associated protein complexes, confirming the observation that LMP1 mainly relies on its transmembrane domains 3 and 4 to interact with FGD4.

### LMP1 directly interacts with FGD4

To further investigate whether LMP1 directly interacted with FGD4, we conducted *in vitro* affinity chromatography assays using GST-FGD4 as bait to precipitate *in vitro*-translated, [^35^S]methionine-labeled LMP1. As shown in Figure 5C, *in vitro*-translated, [^35^S]methionine-labeled LMP1 was precipitated by GST-FGD4, indicating a direct interaction between LMP1 and FGD4.

To buttress the direct interaction between LMP1 and FGD4 within live cells, we carried out an advanced bioluminescence resonance energy transfer (BRET^2^) assay using NPC cells. This technology uses *Renilla* luciferase (Rluc) as the donor molecule and a GFP^2^ as the acceptor molecule in an assay analogous to fluorescence resonance energy transfer (FRET) but without the need for the use of an excitation light source. As shown in Figure 5D, the BRET signal was evident in cells co-expressing Rluc-LMP1 and GFP^2^-FGD4, but not in the controls (Rluc/GFP^2^, Rluc-LMP1/GFP^2^, and Rluc/GFP^2^-FGD4; *P* = 0.005, 0.016, and 0.004, respectively, vs. Rluc-LMP1/GFP^2^-FGD4). Moreover, expression of Flag-LMP1 in combination with Rluc-LMP1/GFP^2^-FGD4 competitively decreased the BRET ratio (*P* = 0.004; paired *t*-test), providing evidence for specificity.

### LMP1 transmembrane domains are required for its co-localization with FGD4, the redistribution of actin, and the promotion of NPC cell motility

**Figure 6:**
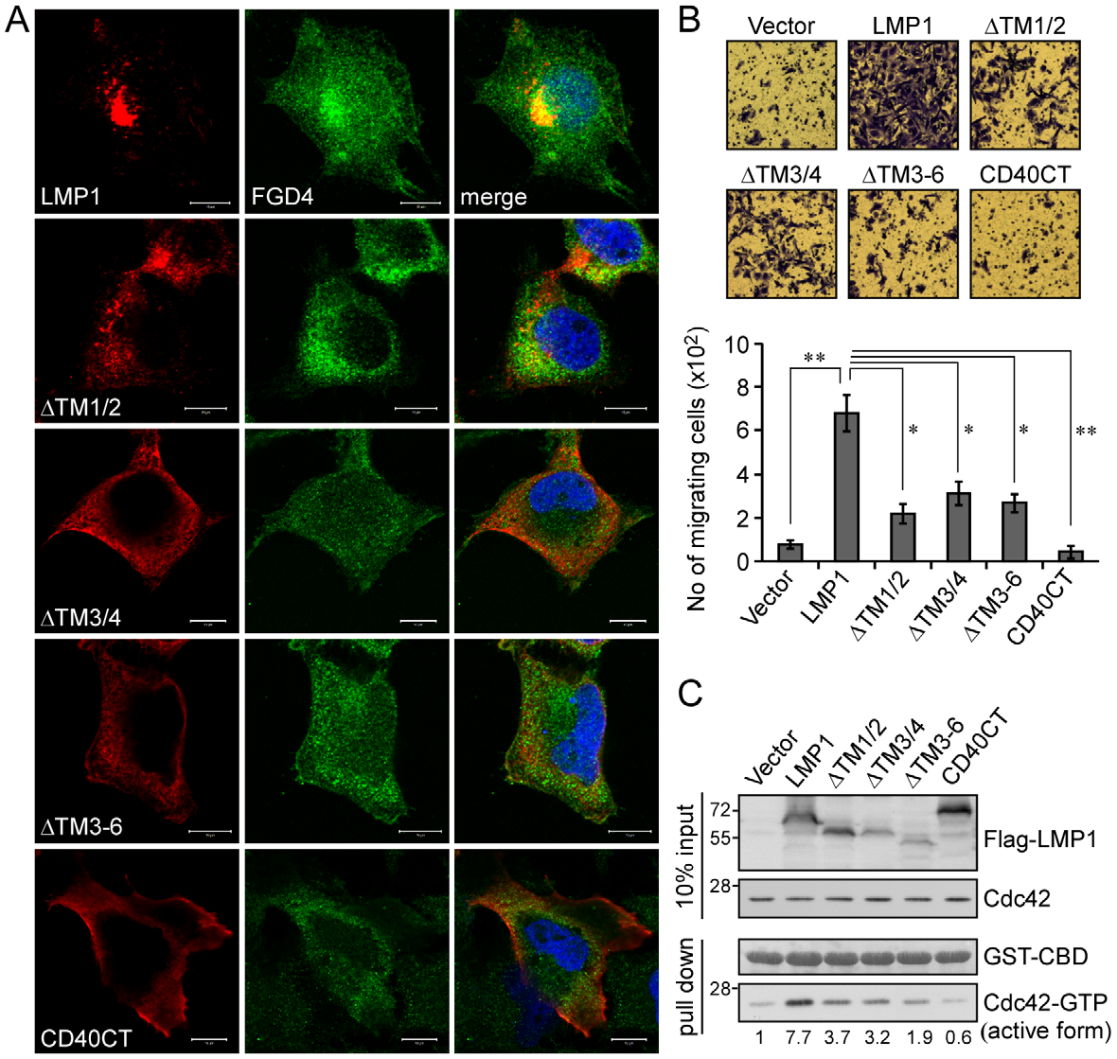
Deletion of the transmembrane domains of LMP1 impairs its ability to co-localize with FGD4 and to promote cell motility. (A) Co-localization of LMP1 with FGD4. NPC-TW02 cells grown on coverslips were transfected with 0.5 µg of expression plasmid for Flag-LMP1 or its truncated or chimeric forms. Cells were fixed after a 24-h incubation and a following 6-h serum starvation, and then co-stained with primary anti-FGD4 and anti-Flag antibodies and secondary FITC-conjugated (green) and TRITC-conjugated (red) antibodies, respectively. Nuclei were identified by DAPI staining (blue). Images were acquired using a ZEISS LSM510 confocal microscope as detailed in Materials and Methods. Scale bars, 10 µm. (B) Induction of cell motility by LMP1. NPC-TW02 cells were transfected with the plasmid for Flag-LMP1 or its truncated or chimeric forms. After 24 h, the cells were re-seeded for transwell migration assays as detailed in Materials and Methods. Images of migrating cells in each experiment were acquired at 200× magnification. The number of migrating cells was counted and presented as the mean ± SD of four independent experiments (*P<0.01, **P<0.005; paired *t*-test). (C) The level of active Cdc42 under each condition was determined by GST-CBD pull-down assays of cell lysates prepared from a portion of cells used in transwell migration assays, as described above. The amounts of GST-CBD were determined by Ponceau S staining.

To investigate whether LMP1 affected the localization of FGD4 through protein-protein interaction, we performed subcellular fractionation using postnuclear extracts of NPC cells expressing LMP1 or the ΔTM3–6 form (Figure 5E), and performed immunofluorescence staining of FGD4 using NPC cells expressing various forms of LMP1 (Figure 6A). As shown in Figure 6A, LMP1 appeared to recruit a fraction of FGD4 to the perinuclear regions (Golgi apparatus) where LMP1 was localized, compared to a primary cytoplasmic localization of FGD4 in control vector-transfected cells (data not shown). In contrast, replacement (CD40CT) or deletion of the transmembrane domains (in particular ΔTM3/4 and ΔTM3–6) of LMP1 substantially impaired its co-localization with FGD4. Moreover, expression of full-length LMP1 led to FGD4 redistribution from fractions 2–5 to fractions 2–9 compared with the vector-expressing cells (Figure 5E); however, expression of the ΔTM3–6 form appeared not to result in this redistribution, suggesting that LMP1 interaction with FGD4 affects the intracellular distribution of FGD4. Concomitantly, LMP1 expression led to a notable redistribution of β-actin (a component of F-actin) from fractions 1–18 to fractions 1–28 (Figure 5E), implying a rearrangement of actin filaments. In contrast, expression of the ΔTM3–6 had no effect on this event, agreeable with the immunofluorescence staining data in Figure 2D. This redistribution was not due to a general alteration of protein localization, because the distribution of caveolin-1 (CAV1) was comparable in all examined cells. While efforts to detect traces of Cdc42 in all fractions were not successful, these data functionally linked the LMP1-FGD4 interaction with actin redistribution, suggesting that LMP1 induces actin rearrangement by enhancing FGD4 activity toward Cdc42.

To investigate whether the above events contributed to cell motility, we conducted transwell migration assays using NPC cells expressing various forms of LMP1. As shown in Figure 6B, LMP1 expression clearly induced cell motility compared with the vector control (*P*<0.001; paired *t*-test); however, deletion of the transmembrane domains apparently impaired this ability of LMP1 (*P*<0.01; paired *t*-test). Moreover, the CD40CT chimera failed to induce cell motility compared with the vector control (*P*<0.005; paired *t*-test), correlating with its eliminated activation of Cdc42 (a 0.6-fold increase; Figure 6C).

### FGD4 and Cdc42 coordinate for LMP1-induced actin rearrangement and cell motility

**Figure 7:**
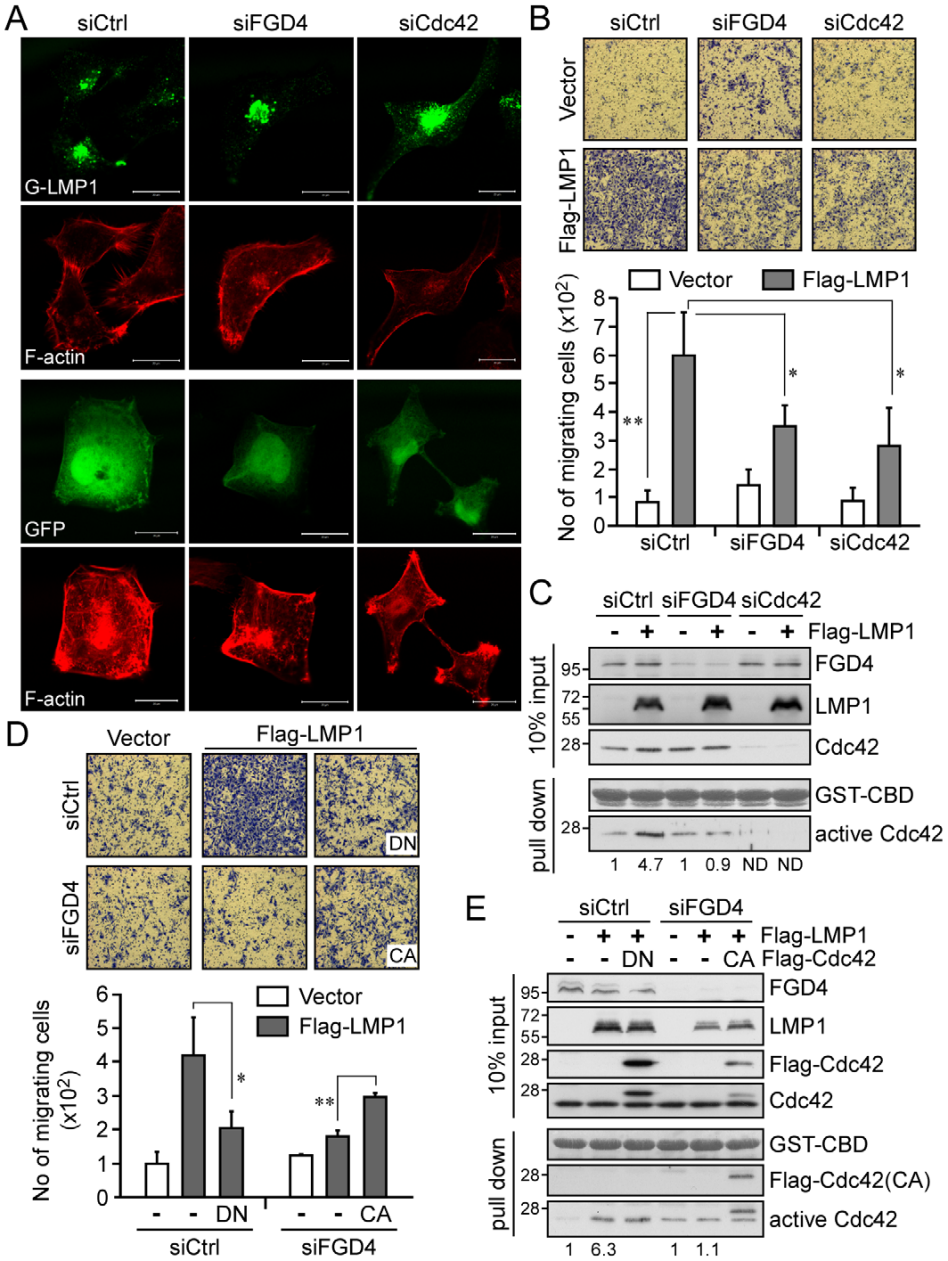
The action of the LMP1-FGD4-Cdc42 axis in NPC cells leads to actin rearrangement and increased cell motility. (A) NPC-TW02 cells grown on coverslips were co-transfected with the indicated siRNA duplexes plus pEGFP-LMP1 or pEGFP vector, and then incubated for 48 h. Following a 6-h serum starvation, cells were fixed and stained with TRITC-conjugated phalloidin to examine actin filament (F-actin) arrangement. Images were acquired using a ZEISS LSM510 confocal microscope. Scale bar, 20 µm. (B) NPC-TW02 cells were co-transfected with the indicated siRNA duplexes and a Flag-LMP1 expression plasmid or empty vector. After 24 h, the cells were re-seeded for transwell migration assays as detailed in Materials and Methods. Images of migrating cells in each experiment were acquired at 200× magnification. The number of migrating cells was counted and presented as the mean ± SD of four independent experiments (*P<0.001, **P<0.01; paired *t*-test). (C) The level of active Cdc42 under each condition was determined by GST-CBD pull-down assay of cell lysates prepared from a portion of cells used in transwell migration assays, as described above. ND, not detectable. (D) NPC-TW02 cells were co-transfected with control or FGD4 siRNA plus a Flag-LMP1 expression plasmid or empty vector, with and without co-transfection of an expression plasmid for Flag-Cdc42DN (DN) or Flag-Cdc42CA (CA). At 24 h post-transfection, the cells were re-seeded for transwell migration assays. Images of migrating cells in each experiment were acquired at 200× magnification. The number of migrating cells was counted and presented as the mean ± SD of three independent experiments (*P<0.05, **P<0.01; paired *t*-test). (E) The level of active Cdc42 under each condition was determined by GST-CBD pull-down assay of cell lysates prepared from a portion of cells used in transwell migration assays, as described above.

To verify the requirement of FGD4 and Cdc42 in LMP1-induced actin rearrangement, we used RNA interference to deplete FGD4 or Cdc42 from NPC cells, followed by expression of GFP-LMP1 and staining for F-actin. As shown in Figure 7A, expression of GFP-LMP1 but not GFP alone in control siRNA (siCtrl)-treated NPC cells resulted in filopodia formation at the plasma membrane. In contrast, knockdown of either FGD4 or Cdc42 led to the disappearance of such actin substructures resulting from GFP-LMP1 expression, demonstrating the equivalent roles of FGD4 and Cdc42 in LMP1-induced actin rearrangement.

To investigate whether the above events result in increased cell motility, we next performed transwell migration assays using NPC cells co-expressing Flag-LMP1 and siRNA specific for FGD4 or Cdc42. The data revealed that, compared to vector-transfected cells, expression of LMP1 in control siRNA (siCtrl)-treated cells increased cell motility (Figure 7B, *P* = 0.0001). This LMP1-induced cell motility was reduced by approximately 50% by knockdown of either FGD4 (*P* = 0.008) or Cdc42 (*P* = 0.005; Figure 7B). This reduced cell motility was associated with a decreased level of LMP1 activation of Cdc42; knockdown of FGD4 decreased the fold-increase in active Cdc42 from 4.7 to 0.9, and knockdown of Cdc42 decreased active Cdc42 to undetectable levels (Figure 7C). Despite its specificity toward Cdc42, FGD4 has also been shown to indirectly promote Rac1 activation [44]. To clarify whether Rac1 activation was involved in LMP1-induced cell motility, we assessed the level of active Rac1 using NPC cells prepared from similar experiments as described above. The results revealed that neither knockdown of FGD4 nor reconstitution of FGD4 can affect the activation of Rac1 in NPC cells (Figure S5A and S5B). In addition, knockdown of Cdc42 had no effect on the activation of Rac1 or RhoA (Figure S5C). Furthermore, neither knockdown of Rac1 nor knockdown of RhoA can affect the LMP1-induced cell motility (Figure S5D and S5E). Taken together, these data conclusively demonstrated that Rac1 and RhoA are not involved in LMP1-induced cell migration of NPC cells.

To buttress the requirement of Cdc42 activity for the LMP1-induced cell motility, we conducted the transwell assays similar to those used in Figure 7B, except we co-expressed Flag-LMP1 with Flag-Cdc42DN (a dominant negative mutant) in control siRNA-treated cells to block Cdc42 activity, or co-expressed Flag-LMP1 with Flag-Cdc42CA in FGD4 siRNA-treated cells to bypass the effect of FGD4 depletion. As shown in Figure 7D, co-expression of LMP1 with Cdc42DN reduced LMP1-induced cell motility by approximately 50% (*P* = 0.02; paired *t*-test), a reduction reminiscent of the effect of FGD4 depletion (Figure 7B). Conversely, co-expression of LMP1 with Cdc42CA partially reversed the reduction in cell motility caused by FGD4 depletion (Figure 7D, *P* = 0.004), revealing that active Cdc42 was responsible for mediating the cell migration triggered by the LMP1-FGD4 axis.

### Both LMP1 and FGD4 are expressed in NPC tissues

**Figure 8:**
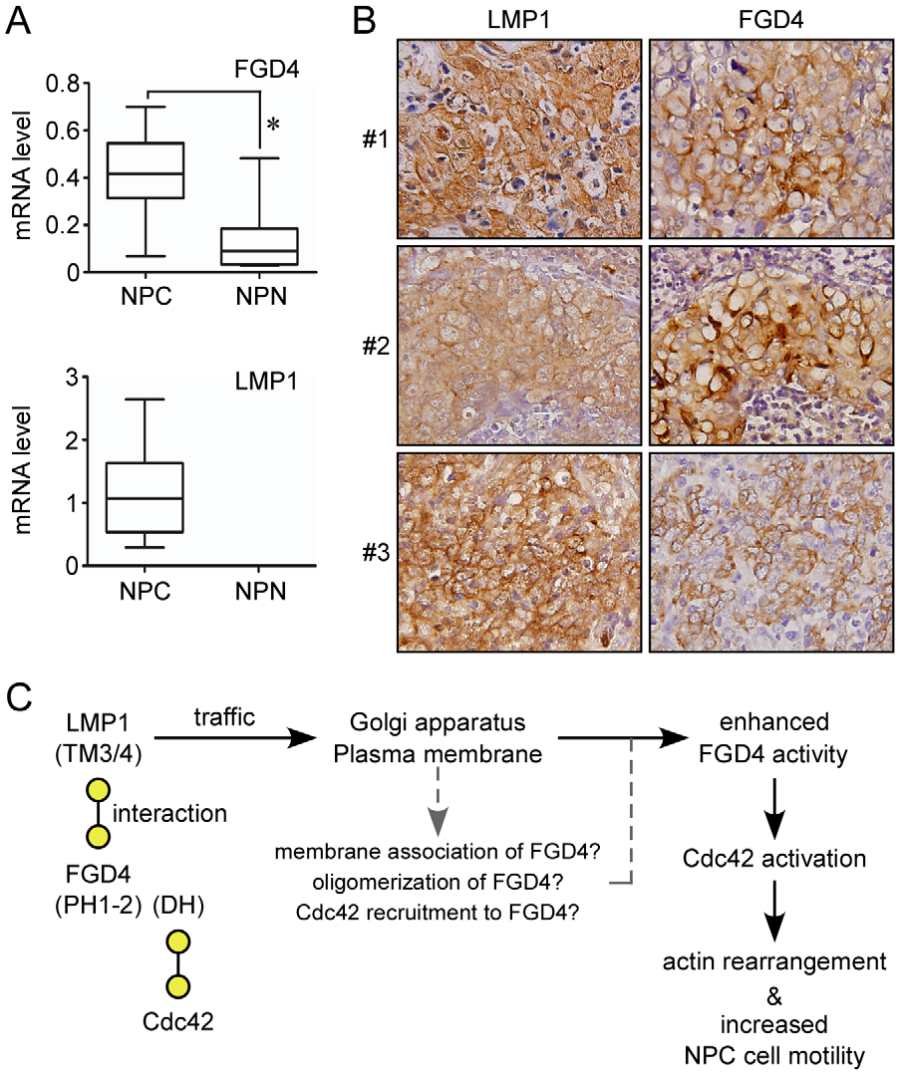
Expressions of LMP1 and FGD4 in NPC tissues. (A) Elevated FGD4 mRNA levels in NPC specimens compared with normal nasopharyngeal tissues. mRNA was isolated from specimens of 13 NPC patients (NPC) and 14 control individuals with nasosinusitis (NPN). Each mRNA sample was then reverse transcribed and analyzed by quantitative RT-PCR using specific primer sets. The expression levels of FGD4 or LMP1 in each specimen were determined by normalizing the reading of FGD4 or LMP1 to that of COL4A6 (Collagen, type IV, alpha), an internal control that showed a consistent expression level among specimens. The data were analyzed by Box-plot analyses. The box indicates the 25th and 75th percentiles of the data range; the middle line indicates the median (*P = 0.0005; two-tailed Mann Whitney test). (B) Immunohistochemical staining for LMP1 and FGD4 in formalin-fixed, paraffin-embedded consecutive NPC tissue sections. Predominant expression of LMP1 and FGD4 was detected at the cell membrane. The images shown are representative from three cases and were acquired at 400× magnification. (C) Model for the action of the LMP1-FGD4-Cdc42 axis in NPC cells. The viral oncoprotein LMP1 interacts with FGD4, a Cdc42 GEF, mainly through the transmembrane domains 3 and 4 (TM3/4) of LMP1 and the PH1-to-PH2 domains (PH1-2) of FGD4. During the intracellular trafficking of LMP1 to the lipid-rich compartments (the Golgi apparatus and the plasma membrane), LMP1 interaction with FGD4 may promote membrane association, oligomerization, and/or conformation change of FGD4, leading to an enhanced FGD4 activity toward Cdc42. The LMP1-involved membrane association of FGD4 may also increase the chance for FGD4 to recruit Cdc42 (via the FGD4 DH domain) that is targeted to the membrane by independent mechanisms, thereby inducing Cdc42 activation. Once activated, active Cdc42 subsequently binds downstream effectors that are involved in regulating the arrangement of actin filament, ultimately contributing to increased cell motility of NPC cells.

To explore the physiological relevance of LMP1 and FGD4 in NPC, we assessed LMP1 and FGD4 expression in NPC specimens. We first performed quantitative RT-PCR analyses using mRNAs isolated from specimens from 13 NPC patients and 14 controls. As shown in Figure 8A, LMP1 mRNA expression was exclusively detected in NPC specimens. Moreover, FGD4 mRNA was expressed at a higher level in NPC specimens than in controls (0.422±0.173 vs. 0.131±0.135; *P* = 0.0005; two-tailed Mann Whitney test); however, no correlation was observed between the levels of FGD4 and LMP1 mRNA (*P* = 1; Spearman test). To detect FGD4 and LMP1 proteins in NPC specimens, we next conducted immunohistochemical staining for FGD4 and LMP1 on consecutive NPC tissue sections from 48 NPC cases. Among them, both FGD4 and LMP1 were detectable in 29 cases and no correlation was shown between the levels of FGD4 and LMP1 (*P* = 0.85; Spearman test). Despite this, it was notable that LMP1 and FGD4 exhibited similar staining patterns, prominently at the cell membrane, as shown in three representative cases (Figure 8B). These data provide a potential physiological relevance for the LMP1-FGD4 interaction in NPC tissues.

Collectively, our data reveal that LMP1 induces Cdc42 activation by directly binding to FGD4, promoting actin rearrangement and, ultimately, cell migration.

## Discussion

Although Puls *et al.* have previously linked Cdc42 activation to LMP1-induced actin rearrangement in fibroblasts [17], the molecular basis of Cdc42 activation by LMP1 and its role in EBV-associated malignancy remained to be elucidated. Here, we demonstrated that LMP1 induces Cdc42 activation in NPC cells via a direct interaction with the Cdc42-specific GEF, FGD4, leading to actin remodeling and increased cell motility. We further verified the expression of LMP1 and FGD4 in NPC specimens, not only providing support for the physiological relevance of this mechanism but also linking FGD4 to tumorigenesis for the first time.

Pathogenic microbes, including viruses, commonly hijack the host cell processes, such as cytoskeleton reorganization, to benefit their own survival in aspects of attachment, entry into cells, movement within and between cells, as well as vacuole formation and remodeling [45], [46]. In addition to LMP1, several viral proteins have been documented to affect the function of Rho protein, which is highly involved in the regulation of cytoskeleton organization. For instance, the E6 oncoprotein of high-risk human papilloma virus type 16 interacts with a binding partner of a GEF (ARHGEF16) to coordinate Cdc42 activation [47], and the Nef protein of human immunodeficiency virus type 1 recruits the GEF Vav1 into plasma membrane microdomains, where it associates with and activates Cdc42/PAK2 (p21-activated kinase 2) [48]. To our knowledge, our evidence that LMP1 elicits Cdc42 activation via direct binding to a Cdc42-specific GEF is the first such demonstration for a viral oncoprotein. It has been proposed that FGD4 is targeted to a preexisting specific actin structure through its FAB domain [35]. LMP1 interaction with FGD4 likely promotes recruitment of FGD4 to the sites where LMP1 is present (Figure 5E and 6A) and thereby elicits Cdc42 activation in the vicinity of the actin structure associated with FGD4 (Figures 2D and S2). This process ultimately results in spatial reorganization of the actin cytoskeleton and regulates cell morphogenesis and cell motility [35], [49], in line with our observation that LMP1 induces the formation of filopodia at the cell surface (Figures 2D and 7A) and promotes cell migration via FGD4/Cdc42 (Figure 7B–7E).

In this study, we demonstrated that LMP1 directly interacts with FGD4 (Figure 5C and 5D) and enhances FGD4 activity toward Cdc42 (Figure 4F). The LMP1-FGD4 interaction requires the transmembrane domains 3 and 4 of LMP1 and the PH1-to-PH2 domains (phosphoinositide-binding domains) of FGD4 (Figures 4E, 4F, 5A, and 5B), indicating that the membrane/lipid association of both proteins may allow or enhance their interaction. We have previously identified that upon synthesis in the endoplasmic reticulum (ER), LMP1, through its transmembrane domains 3–6, interacts with PRA1 (the prenylated Rab acceptor 1) for transport from the ER to the Golgi apparatus [50], an intracellular compartment where LMP1 primarily induces signaling pathways [50], [51]. Deletion of the LMP1 transmembrane domains (in particular 3–6) or knockdown of PRA1 leads to LMP1 retention in the pre-Golgi compartment (Figures 2D, 5E, 6A, and S2B; [50]) concomitant with a reduction of Cdc42 activation (Figures 2B, 2C, and 6C; data not shown), implying that the proper localization of LMP1 is needed for its full activation of Cdc42. These data elicit an argument that the impaired FGD4 interaction of the truncated LMP1 (ΔTM3/4 and ΔTM3–6) actually arises from the impaired trafficking process instead of deletion of specific transmembrane domains. Here we propose that LMP1 interacts with FGD4 via the transmembrane domains 3 and 4, and subsequently recruits and/or enhances FGD4 association with the membrane during the Golgi-directed trafficking of LMP1 (Figure 8C). This model is supported by several lines of data as the following. First, expression of LMP1 but not its truncated form (in particular ΔTM3–6) leads to FGD4 redistribution toward the LMP1-containing fractions as demonstrated by subcellular fractionation (Figure 5E) and immunofluorescence staining (Figure 6A). These data suggest that LMP1 interaction with FGD4 indeed occurs prior to LMP1 localization to specific compartments. Second, the short N-terminus and the transmembrane domains 1 and 2 of LMP1 have been shown to be required for targeting of LMP1 to the lipid raft [52], [53]. Deletion of these domains (denote ΔNT and ΔTM1/2) also impedes the Golgi-directed trafficking of LMP1 (data not shown; Figures 2D and 6A) but does not abolish LMP1 interaction with FGD4 (Figure 5A and 5B), revealing that this interaction is not restricted at certain compartments but rather relies on the transmembrane domains 3 and 4 of LMP1. Agreeably, the CD40CT chimera fails to interact with FGD4 (Figures 4F, 5A, and 5B) despite its intact membrane localization (Figures 2D and 6A).

Like some GEFs whose activation is stimulated by protein-protein interaction [54]–[56], it is assumed that FGD4 activation is potentiated by interaction with LMP1 or self-oligomerization (Figure S4C). Further localization of LMP1 and FGD4 to lipid-rich compartments (the Golgi apparatus and the plasma membrane) likely strengthens the LMP1-FGD4 interaction and the membrane association of FGD4, leading to an enhanced FGD4 activity toward Cdc42 (the model in Figure 8C). Conceivably, the lipid in the membrane may enhance stimulation of FGD4 activities. In line with this, deletion of the PH1-to-PH2 domains of FGD4 substantially impairs its activation of Cdc42 (Figure 4C), indicating that membrane/lipid association is involved in the regulation of FGD4 activity. As the truncated LMP1 (ΔTM3/4 and ΔTM3–6) still retains ∼30% activation of Cdc42, we speculate that the targeting of LMP1 to the lipid raft (via the N-terminus and the transmembrane domains 1 and 2) may indirectly assist FGD4 to associate with the lipid/membrane. Further investigation will be needed to clarify the mechanisms in greater details. Importantly, LMP1-induced Cdc42 activation can be blunted by knockdown of FGD4 (Figure 3) or overexpression of functionally impaired forms of FGD4 (Figure 4D), indicating that LMP1 acts upstream of FGD4 rather than in parallel with it to induce Cdc42 activation. We propose that the functionally impaired forms of FGD4 (ΔDH and PH1-2) inhibit LMP1-induced Cdc42 activation by competitively impeding the interaction between LMP1 and full-length FGD4 (Table S1). Intriguingly, the FAB-DH form of FGD4, which interacts poorly with LMP1, could still inhibit LMP1-induced Cdc42 activation, probably by competing with full-length FGD4 for access to Cdc42. Apart from this, phosphatidylinositol 3-kinase (PI3K) has been linked to the translocation of FGD4 during infection of the enteric parasite *Cryptosporidium parvum* [57]. In this case, *C. parvum* infection induces recruitment of FGD4 to the host cell-parasite interface; this process, which results in Cdc42 activation, is dependent on PI3K and is required for *C. parvum*-induced actin remodeling and cellular invasion [57]. Although it has been demonstrated that LMP1 is able to act through its C-terminus to activate PI3K [58], our study found that this region is dispensable for LMP1 activation of Cdc42 (Figure 2B). Moreover, treatment of LMP1-expressing cells with the PI3K inhibitors, wortmannin and LY294002, did not block the LMP1-induced Cdc42 activation (data not shown), indicating that the LMP1-associated functional regulation of FGD4 involves the LMP1-FGD4 interaction instead of PI3K activity.

We noted that LMP1, together with FGD4, is expressed heterogeneously at the plasma membrane as well as the Golgi apparatus (Figure 6A) and induces Cdc42 activation at these sites (Figure S2A). It has been suggested that restricted localization of active Cdc42 is important for its distinct functions [59], suggesting the possibility that LMP1 induction of Cdc42 activation at the plasma membrane and the Golgi apparatus serves distinct purposes. Given that Cdc42 also controls the intracellular protein trafficking, including the Golgi-to-ER retrograde transport [60], [61] and protein exit from the trans-Golgi network [19], [62], it is conceivable that the LMP1-FGD4-Cdc42 cascade may have a regulatory role in intracellular protein transport apart from cell migration. We speculate that LMP1-induced Cdc42 activation may attenuate Golgi-to-ER retrograde protein transport [60], promoting LMP1 retention at the Golgi apparatus and sustaining LMP1-mediated signaling. Further investigation will be needed to dissect the interplays between LMP1 and FGD4 within distinct compartments and elucidate how their functions are coordinated to affect cellular processes.

Although FGD4 has been implicated in neural development [29], [63], [64], we here delineate a potential role for FGD4 in NPC progression that is associated with LMP1. We verified the expression of LMP1 and FGD4 in NPC tissues at both mRNA and protein levels using the quantitative RT-PCR and immunohistochemistry, respectively (Figure 8A and 8B). Intriguingly, FGD4 mRNA expression appears to be elevated in cancerous tissues compared to the normal controls (Figure 8A), although the underlying mechanism remains to be identified. In any case, it is conceivable that higher levels of FGD4 in NPC tissues lead to an increase in FGD4 function. In addition, it was recently shown that FGD1, which is functionally related to FGD4, is up-regulated in human prostate and breast cancer, and regulates cancer cell invasion by modulating Cdc42 activation in a cell model [65]. Accordingly, our findings highlight the importance of elucidating the mechanism by which FGD4 and its related proteins are dysregulated in tumor development.

## Materials and Methods

### Ethics statement

This research followed the tenets of the Declaration of Helsinki and all subjects signed an informed consent approved by Institutional Review Board of Chang Gung Memorial Hospital before their participation in this study and for the use of tissue samples collected before treatment.

### Cell culture

NPC-TW01, -TW02, -TW04 and -TW06 cell lines, which had been established using NPC biopsy specimens collected from four NPC patients, respectively [66], [67], were cultured in Dulbecco's modified Eagle's medium (DMEM) supplemented with 10% fetal bovine serum (FBS) at 37°C in a humidified 5% CO_2_ environment. The inducible LMP1-expressing 293 cell line (293 Tet-On), generated previously [50], was grown on 1% collagen-coated dishes and maintained in DMEM supplemented with 10% FBS, 100 µg/ml G418, and 50 µg/ml hygromycin. The human embryonic kidney cell line HEK293, obtained from American Type Culture Collection (ATCC; CRL 1573), was grown on 1% collagen-coated dishes and cultured in DMEM containing 10% equine serum. The nasopharyngeal epithelium cell line (NP69) and a stable LMP1-expressing NP69 cell line (NP69-LMP1) were generous gifts from Dr. Sai-Wah Tsao (University of Hong Kong, China). The growth medium used for NP69 and NP69-LMP1 cells has been previously described in detail [68]. Unless specified, all the reagents were purchased from Invitrogen (Carlsbad, CA, USA).

### Clinical specimens

Freshly frozen biopsied tissues from 13 NPC patients and 14 control individuals with nasosinusitis, and slide-mounted consecutive NPC tissue sections from 48 NPC patients were collected at Chang Gung Memorial Hospital (Lin-Kou, Taiwan). Clinical data for the NPC patients are presented in Table S2.

### Antibodies

The anti-LMP1 monoclonal antibody (S12) was affinity purified from a hybridoma. Mouse anti-Flag (M2) and anti-HA (12CA5) antibodies were purchased from Sigma-Aldrich (St. Louis, MO, USA); a mouse anti-Myc tag (9E10) antibody was purchased from Cell Signaling Technology (Danvers, MA, USA); mouse anti-human Cdc42 and anti-Rac1 antibodies were purchased from BD Transduction Laboratories (BD Biosciences, San Jose, CA, USA); a mouse anti-human RhoA antibody was purchased from Santa Cruz Biotechnologies, Inc. (Santa Cruz, CA, USA). Rabbit antibodies against human DOCK9, intersectin-1, FGD3, and CAV1 were purchased from Santa Cruz Biotechnologies; rabbit anti-human FGD1 antibody was purchased from GeneTex (Irvine, CA, USA); rabbit anti-human FGD4 antibody was purchased from both Novus Biologicals (Littleton, CO, USA) and GeneTex (Irvine, CA, USA). Fluorescein isothiocyanate (FITC)-, tetramethylrhodamine isothiocyanate (TRITC)-, and horseradish peroxidase (HRP)-conjugated secondary antibodies were purchased from BD Transduction Laboratories.

### Plasmid construction

N-terminally Flag-tagged LMP1 as well as its truncated derivatives were generated by ligation of PCR-amplified DNA fragments to *Hind*III/*Bam*HI-treated pCMV2-Flag (Kodak) as described previously [50]. GFP-tagged LMP1 was generated by ligation of DNA fragments to *Hind*III/*Bam*HI-treated pEGFP-C3 (BD Biosciences). Full-length and truncated FGD4 constructs were generated by PCR using human FGD4 cDNA as a template; the resulting DNA fragments were subsequently inserted into pCMV2-Flag at the *Eco*R1/*Xba*I sites or into pCMV-Myc (BD Biosciences) at the *Kpn*I/*Xba*I sites. Glutathione S-transferase (GST)-tagged full-length FGD4 construct was created by ligating the respective PCR-amplified DNA fragments into *Eco*RI/*Xho*I-treated pGEX 4T.1 (BD Biosciences). Plasmids encoding the GST fusion proteins, GST-CBD (containing the active Cdc42-binding domain of Wiskott-Aldrich syndromewith protein, WASP; aa 201–321), GST-PBD (containing the active Rac1-binding domain of PAK1; aa 70–132), and GST-RBD (containing the active RhoA-binding domain of Rhotekin; aa 7–113) were gifts from Dr. Jacques Bertoglio (INSERM U461, Faculté de Pharmacie-Paris Sud, Chatenay-Malabry, France). pEGFP-CBD was generated by subcloning the DNA fragments encoding the CBD of WASP into pEGFP-C3. Flag-tagged, constitutively active (CA) and dominant-negative (DN) versions of Cdc42, Cdc42L61 and Cdc42N17, respectively, were generated by site-directed mutagenesis using primers bearing the desired sequence changes. The DNA fragments were subsequently inserted into pCMV2-Flag at *EcoR*I/*Bam*HI sites. For BRET^2^ assays, N-terminally Rluc-tagged LMP1 and GFP^2^-tagged FGD4 were generated by ligation of PCR-amplified DNA fragments into *Kpn*I/*Bam*HI-treated pRluc(h)-C2 and *Kpn*I/*Xba*I-treated pGFP^2^-C1 vectors (PerkinElmer Life and Analytical Sciences, MA, USA), respectively. Primer sequences used for cloning are provided in Table S3.

### Cell transfection and RNA interference

Unless specified, plasmid transfections were carried out using Lipofectamine (Invitrogen), according to the manufacturer's instructions. Cells were incubated for 24 h prior to further treatments. For RNA interference, cells were co-transfected with 25 nM siRNAs as a set of four duplexes (SMARTpool) directed against a specific gene, together with plasmids indicated elsewhere, using Lipofectamine 2000 (Invitrogen) according to the manufacturer's instructions. Cells transfected with non-targeting duplexes (siCtrl) were used as a negative control. Cells were incubated for 48 h before further treatments, and the efficiency of gene silencing was estimated by quantitative RT-PCR and Western blotting. All siRNAs were purchased from Dharmacon (CO, USA) except Rac1 siRNAs, which were purchased from Invitrogen.

### Quantitative RT-PCR

Total RNA was purified using TRIzol (Invitrogen) and 1 µg of each sample was reverse transcribed using ImProm-II and Oligo(dT)_15_ primers (Promega). The resulting cDNAs were analyzed using the FastStart DNA Master SYBR Green I reagent (Roche, Germany) on a LightCycler (Roche), according to the manufacturer's instructions. The reactions were incubated at 95°C for 10 min, followed by 45 cycles of 95°C for 10 s, 60°C for 5 s and 72°C for 10 s. Primer sequences used in RT-PCR experiments are presented in Table S4. The levels of individual target mRNAs were normalized to the level of glyceraldehyde-3-phosphate dehydrogenase (GADPH) mRNA in each sample.

### Purification of GST-fusion proteins

pGEX construct-transformed *Escherichia. coli* (strain BL21) were grown to mid-exponential phase, induced for 4 h with 0.5 mM isopropylthiogalactoside and lysed by sonicating in PBST buffer (1× phosphate-buffered saline [PBS] with 2 mM EDTA, 0.1% β-mercaptoethanol, 0.2 mM phenylmethylsulfonyl fluoride [PMSF], and 5 mM benzamidine). The GST fusion proteins were purified from bacterial lysates by incubation with glutathione-coupled Sepharose beads (Amersham Biosciences).

### Precipitation of active Cdc42, Rac1, and RhoA

The cellular level of the GTP-bound form of Cdc42 was determined using the GST-CBD pull-down assays. Briefly, at given incubation time after transfection, cells were cultured in serum-free media for an additional 6 h and lysed in Nonidet P-40 (NP-40) lysis buffer (1% NP-40, 20 mM Tris-HCl pH 7.5, 150 mM NaCl, 1 mM Na_3_VO_4_, 5 mM EDTA pH 8.0, 10% glycerol, 10 µg/ml leupeptin, 10 µg/ml aprotinin, 1 mM PMSF). After centrifugation, the resulting lysates (1 mg protein) were incubated with 20 µg of immobilized GST-CBD proteins at 4°C for 1 h. CBD-bound proteins were centrifuged, washed three times in NP-40 lysis buffer, boiled in sodium dodecyl sulfate (SDS) sample buffer and analyzed by Western blotting, as described below, using a Cdc42-specific antibody. GST-PBD and GST-RBD were used for precipitation of active forms of Rac1 and RhoA, respectively, following a similar procedure.

### *In vitro* transcription/translation and *in vitro* affinity chromatography assays

Full-length LMP1 cDNA was constructed and cloned into pcDNA3.1 for *in vitro* transcription/translation. [^35^S]methionine-labeled proteins were generated using the TNT Coupled Reticulocyte Lysate System (Promega, Madison, WI, USA), following the manufacturer's recommendation. Briefly, a reaction mixture (total volume, 100 µl) containing 50 µl of TNT Rabbit Reticulocyte Lysate, 4 µl of TNT Reaction Buffer, 2 µl of TNT T7 RNA Polymerase, 2 µl of Amino Acid Mixture Minus Methionine (1 mM), 2 µl of RNasin Ribonuclease Inhibitor (Promega), 2 µg of pcDNA3.1-LMP1, 4 µl of [^35^S]methionine (10 mCi/ml; Izotop, Hungary), 4 µl of Canine Pancreatic Microsomal Membrances (Promega), and 30 µl of nuclease-free water was incubated at 30°C for 90 min. Equal amounts of the *in vitro*-translated, [^35^S]methionine-labeled LMP1 protein were incubated with 20 µg of GST or GST-FGD4 immobilized on glutathione-Sepharose beads, rotated overnight at 4°C, and then washed five times with NP-40 lysis buffer. The bound LMP1 proteins were resolved by SDS-PAGE on a 10% gel followed by autoradiography.

### Co-immunoprecipitation assays

Cells expressing Flag-tagged and/or Myc-tagged proteins were extracted in 1% NP-40 lysis buffer as described above and fractionated by centrifugation (10,000 g, 15 min at 4°C) to obtain cell lysates. For co-immunoprecipitation, cell lysates (500 µg protein) from NPC cells expressing Flag- or Myc-tagged proteins were incubated with 40 µl of a 50% (w/v) slurry of anti-Flag M2 agarose (Sigma-Aldrich) or with 25 µl of a 50% (w/v) slurry of anti-Myc affinity matrix (Sigma-Aldrich), rotated overnight at 4°C, and then washed five times with NP-40 lysis buffers. For co-immunoprecipitation of endogenous FGD4 and LMP1, cell lysates (1 mg protein) from NPC cells expressing LMP1 were incubated with 2 µg of anti-FGD4 IgG or control IgG together with 20 µl of a 50% (w/v) slurry of protein G (Amersham Biosciences), and further processed using procedures similar to those above. The resulting protein products were eluted with SDS sample buffer and analyzed by Western blotting using appropriate primary and secondary antibodies.

### Western blot analysis

Cells were lysed in 1% NP-40 lysis buffer as described above. Protein concentrations were determined using the Protein Assay Reagent (Bio-Rad, CA, USA), and equal amounts of proteins (30–50 µg/lane) were resolved by SDS-polyacrylamide gel electrophoresis (SDS-PAGE) on 7.5%–12% polyacrylamide gels. The proteins were then electro-transferred onto nitrocellulose (NC) membranes (Amersham Biosciences). After blocking with 5% non-fat powdered milk in TBS, membranes were incubated with the respective primary antibodies overnight at 4°C. Membranes were then incubated with the appropriate HRP-conjugated secondary antibody for 1 h at room temperature. Protein bands were detected using enhanced chemiluminescence reagents (Pierce ECL, Thermo Scientific) and Fuji SuperRx film.

### BRET^2^ assay

NPC-TW01 cells grown on a 10 cm-dish were transfected with plasmids encoding donor *Renilla* luciferase (Rluc)-tagged LMP1 (1 µg) and acceptor GFP^2^-tagged FGD4 (3 µg). Cells were detached at 24 h after transfection and washed with Dulbecco's Phosphate Buffered Saline (D-PBS; Invitrogen) and then resuspended in D-PBS to a final density of approximately 2×10^6^ cells/ml. Approximately 1×10^5^ cells/well were distributed in a 96-well white polystyrene microplate (Conig, NY, USA). The DeepBlueC coelenterazine substrate (PerkinElmer Life and Analytical Sciences) was added to a final concentration of 5 µM, and bioluminescence emission was monitored immediately using a Fluoroskan Ascent FL microplate fluorometer (Thermo Electron Corporation, MA, USA), which allows the sequential integration of signals detected in 410-nm and 515-nm windows. The BRET^2^ ratio is calculated as the following ratio: (emission of transfected cells at 515 nm – emission of non-transfected cells at 515 nm)/(emission of transfected cells at 410 nm – emission of non-transfected cells at 410 nm). The expression level of each fusion protein was analyzed by Western blotting with appropriate antibodies.

### Subcellular fractionation

NPC-TW04 cells grown on a 10-cm dish were transfected with 1 µg of pFlag-LMP1, pFlag-LMP1ΔTM3–6, or pFlag-CMV2 vector and incubated for 24 h. Cells were then homogenized and centrifuged, and the resulting supernatant was layered onto a continuous sucrose gradient (10%–45% sucrose) and centrifuged for 1 h at 55,000 rpm. using an SW55 rotor (Beckman, Fullerton, CA, USA). The fractions were collected manually from the top of the gradient and 30 µl of every other fraction was subjected to Western blot analysis. Details of this assay have been described previously [50], [69].

### Immunofluorescence microscopy

Cells grown on poly-L-lysine-coated coverslides were fixed with 4% formaldehyde, and permeabilized and blocked with 0.1% saponin containing 1% BSA for 20 min at room temperature. For co-staining of Flag-LMP1 and FGD4, cells were incubated with anti-Flag antibody (M2; 1∶200 dilution) and anti-FGD4 antibody (GeneTex; 1∶50 dilution) for 2 h at room temperature, followed by incubation with the respective fluorophore-conjugated secondary antibody for 45 min. For actin filament staining, cells were incubated in TRITC-conjugated phalloidin (50 µg/ml; Sigma-Aldrich) for 1 h after fixation. Nuclei were stained with 4′,6-diamidino-2-phenylindole (DAPI; Sigma-Aldrich). All coverslides were mounted with the Vectashield reagent (Vector Laboratories Inc., CA, USA) and visualized by confocal microscopy using a ZEISS LSM510 META laser-scanning confocal microscope (Carl Zeiss, Germany) with a 63×1.32 NA oil-immersion objective.

### Immunohistochemistry

For detection of LMP1 and FGD4, consecutive slide-mounted NPC sections were first treated with proteinase K at room temperature for 15 min. Endogenous peroxidase activity was inhibited by incubating with 3% H_2_O_2_ (DAKO). Nonspecific binding was blocked with Antibody Diluent and Background Reducing Component (DAKO). Sections were then incubated with anti-FGD4 (GeneTex; 1∶50 dilution) and anti-LMP1 (S12, 1∶15 dilution) antibodies at room temperature for 1 h. After a washing step, a HRP-conjugated secondary antibody was added and sections were incubated at room temperature for 20 min. Tissue sections were then treated with DAB reagent (DAKO); 3,3′-diaminobenzidine tetrahydrochloride was used as a chromogen. All images were acquired on an Olympus BX51 microscope (Olympus, Japan). Expression of LMP1 and FGD4 was evaluated according to the simplified H score system [70], which is based on the percentage of cell staining: 3 (≥90%), 2 (50%–89%), 1 (10%–49%), or 0 (0%–9%), and the intensity of cell staining: 3 (high), 2 (moderate), 1 (low), or 0 (no cell staining). The two scores were multiplied by each other and then divided by three to get the final score.

### Transwell migration assay

The motility of NPC-TW02 cells was evaluated by transwell migration assays using a chemotaxis chamber (Corning, NY, USA). Using calcium phosphate precipitation, NPC-TW02 cells were transfected with 1 µg of plasmid for Flag-LMP1, its transmembrane domain-truncated forms, CD40CT chimera, or empty vector. For knockdown experiments, NPC-TW02 cells were co-transfected with siRNAs (37.5 nM) directed against FGD4 (siFGD4), Cdc42 (siCdc42) or non-targeting duplexes (siCtrl), plus 1 µg of pFlag-LMP1 or pCMV-Flag vector. In a subset of assays, cells were co-transfected with pFlag-LMP1 (0.75 µg) plus pFlag-Cdc42DN or pFlag-Cdc42CA (1.5 µg each). The oligonucleotides were mixed thoroughly in 250 µl of solution A (136.7 mM NaCl, 19.2 mM HEPES, pH 6.95), 2.5 µl of solution B (57.6 mM Na_2_HPO_4_), and 12.5 µl of solution C (2.5 M CaCl_2_). After 30-min incubation at room temperature, the mixtures were added to cells and incubated for 6 h at 37°C. Twenty-four hours after transfection, cells were trypsinized and washed twice with serum-free DMEM, and then resuspended in 100 µl of serum-free DMEM and seeded into the insert chamber (3×10^5^ cells). After a 20-h incubation, cells that had migrated to the opposite side of the insert (immersed in DMEM supplemented with 10% FBS) in the lower well were fixed and stained with crystal violet (1% crystal violet and 5% formaldehyde in 70% ethanol) for 30 min, followed by washing twice with double-distilled H_2_O (ddH_2_O) to remove the background staining. The number of migrating cells was counted in images acquired at 200× magnification for each experiment and analyzed with NIH Image J software.

### Statistical analyses

Quantitative data were presented as means ± SDs for five independent experiments. Significance between groups was calculated using two-tailed paired *t*-tests. For clinical specimens, significance between groups was calculated using two-tailed Mann-Whitney tests. Correlation between groups was analyzed using two-tailed Spearman tests. A *P*-value<0.05 was considered statistically significant.

### Accession numbers

The Entrez Gene ID numbers for genes or proteins described in this study are as follows: 5176215 (LMP1), 121512 (FGD4), 998 (Cdc42), 5879 (Rac1), 387 (RhoA), 60 (β-actin), 2245 (FGD1), 89846 (FGD3), 6453 (Intersectin-1), 23348 (DOCK9), 857 (CAV1), 7124 (TNF), 3552 (IL-1α), 958 (CD40).

## Supporting Information

Figure S1 **No apparent effect of TNF-α and IL-1α on Cdc42 activation in NPC cells.** (A) Neither TNF-α nor IL-1α can induce Cdc42 activation. NPC-TW01 cells cultured for 24 h after seeding were treated with recombinant TNF-α (50 or 100 ng/ml) or with IL-1α (10 ng/ml) for 30 min under a serum-free condition. Cells without cytokine treatment were used as a control. Each cell lysate was then harvested for the GST-CBD pull-down assays to determine the level of active Cdc42. A reduced protein level of IκBα indicates the activation of NF-κB signaling under the indicated treatment. (B) No apparent effect of TNF-α and IL-1α on actin organization. NPC-TW04 cells that had been grown on poly-L-lysine-coated coverslips overnight were treated with TNF-α (100 ng/ml) or with IL-1α (10 ng/ml) for 30 min under a serum starvation condition. Then cells were fixed and permeabilized, followed by subsequent staining with primary anti-p65 and FITC-conjugated secondary antibodies. Cells were co-stained with TRITC-conjugated phalloidin to reveal the actin filaments. Nuclei were identified by DAPI staining. Translocation of p65 from the cytoplasm to the nuclei evidenced the activation of NF-κB signaling. Images were acquired using a ZEISS LSM510 confocal microscope. Scale bars, 20 µm.
(PDF)

Figure S2 **LMP1 appears to induce Cdc42 activation at LMP1-resident sites.** (A) Spatial distribution of active Cdc42 upon LMP1 expression. 293 Tet-On cells that had been grown on poly-L-lysine-coated coverslips overnight were transfected with 1 µg of plasmid encoding an indicator for active Cdc42, EGFP-CBD, and incubated for 24 h with or without Dox (5 µg/ml) induction of LMP1 expression. Following a 6-h serum starvation, cells were fixed with 3.7% formaldehyde and subsequently stained with primary anti-LMP1 (S12) and TRITC-conjugated secondary antibodies. Nuclei were identified by DAPI staining (blue). Images were acquired using a ZEISS LSM510 confocal microscope. Scale bars, 15–17 µm. The insets were acquired at higher magnification (scale bars, 2–5 µm). (B) Co-localization of a portion of Cdc42 with LMP1. NPC-TW04 cells grown on coverslips overnight were transfected with 0.5 µg of plasmid for EGFP-LMP1 or its ΔTM3–6 truncated form and then incubated for 24 h. Following a 6-h serum starvation, cells were fixed and subsequently stained with a primary anti-Cdc42 antibody (P1, Santa Cruz; 1∶50 dilution) and a TRITC-conjugated secondary antibody. Nuclei were identified by DAPI staining (blue). Images were acquired using a ZEISS LSM510 confocal microscope. Scale bars, 20 µm. The insets demonstrated co-localization of a portion of Cdc42 with EGFP-LMP1 (yellow spots) rather than with the ΔTM3–6 form.
(PDF)

Figure S3 **Knockdown of FGD4 in NPC cells and sequence comparison of FGD4 between species.** (A) Knockdown efficiency of the targeted GEFs in NPC cells expressing LMP1 or transfected with empty vector (control). Total RNA isolated from cells of each treatment group was reverse transcribed and analyzed by quantitative RT-PCR using specific primer sets. The knockdown efficiency for each targeted GEF in each treatment is presented as a ratio of the mRNA level of each GEF in the knockdown cells divided by that in the respective control. (B) Sequence comparison of human, rat, and mouse FGD4. Shaded regions represent the conserved functional domains of FGD4. Asterisks (*) indicate identical amino-acid residues.
(PDF)

Figure S4 **Functional characterization of FGD4.** (A) NPC-TW01 cells grown on coverslips overnight were transfected with 0.5 µg of plasmid for Myc-FGD4 or its truncated forms and then incubated for 24 h. Following a 6-h serum starvation, cells were fixed and subsequently stained with a primary anti-Myc antibody (9E10) and a FITC-conjugated secondary antibody. Then the cells were co-stained with TRITC-conjugated phalloidin to indicate the actin filaments. Images were acquired using a ZEISS LSM510 confocal microscope. Scale bars, 20 µm. (B) FGD4 associates with Cdc42 mainly through the DH domain. NPC-TW01 cells were co-transfected with 1 µg of expression plasmid for Flag-tagged Cdc42 and 1 µg of expression plasmid for Myc-tagged full-length or truncated FGD4. At 24 h post-transfection, the cells were lysed and the resulting cell lysates were analyzed by co-immunoprecipitation using an anti-Flag affinity matrix. The precipitated proteins as well as unprecipitated lysates (input) were analyzed by Western blotting with anti-Flag and anti-Myc antibodies. (C) Self-association of FGD4. NPC-TW01 cells were co-transfected with 1 µg of expression plasmid for Flag-FGD4 and 1 µg of expression plasmid for Myc-FGD4 or its truncated forms. At 24 h post-transfection, the cells were lysed and the resulting cell lysates were subjected to co-immunoprecipitation assays using an anti-Flag affinity matrix, as described above.
(PDF)

Figure S5 **Rac1 and RhoA are not involved in the NPC cell motility mediated by the LMP1-FGD4-Cdc42 axis.** (A) Knockdown or (B) re-introduction of FGD4 has no effect on Rac1 activation. NPC-TW04 cells were co-transfected with 25 µM control or FGD4 siRNA and 1 µg of expression plasmid for Flag-LMP1 or empty vector. In the case of re-introduction, 2 µg of Myc-FGD4 expression plasmids or Myc vector were added into the transfection. After 48-h incubation and a following 6-h serum starvation, cells were lysed for GST-PBD pull-down assays and analyzed for the level of active Rac1. (C) LMP1-induced Cdc42 activation is not associated with Rac1 or RhoA activation. NPC-TW02 cells were co-transfected with control or Cdc42 siRNA duplexes and 1 µg of expression plasmid for Flag-LMP1 or empty vector. After 48-h incubation and a following 6-h serum starvation, cells were lysed for GST-PBD and GST-RBD pull-down assays to analyze the levels of active Rac1 and active RhoA, respectively. (D) Rac1 and RhoA are not involved in LMP1-induced cell migration. NPC-TW02 cells were co-transfected with control, Rac1 or RhoA siRNA duplexes plus a plasmid for Flag-LMP1 or empty vector. Cells were then re-seeded for transwell migration assays as detailed above. (E) The knockdown efficiency was confirmed by Western blot analysis of a portion of cells used in transwell migration assays with anti-Rac1 and anti-RhoA antibodies.
(PDF)

Table S1 **Functional characterization of FGD4 domains and their respective effects on LMP1-mediated Cdc42 activation.**
(PDF)

Table S2 **Clinical information for the nasopharyngeal biopsies used in the quantitative RT-PCR and immunohistochemistry analyses.**
(PDF)

Table S3 **Primer sets for generating expression vectors.**
(PDF)

Table S4 **Primer sets for quantitative RT-PCR.**
(PDF)
