# Supplementary figures and images for: Epstein-Barr Virus-Encoded LMP1 Interacts with FGD4 to Activate Cdc42 and Thereby Promote Migration of Nasopharyngeal Carcinoma Cells
Source: PLoS Pathog. 2012 May 10;8(5):e1002690. doi: 10.1371/journal.ppat.1002690 (PMC3349753; doi:10.1371/journal.ppat.1002690)

A

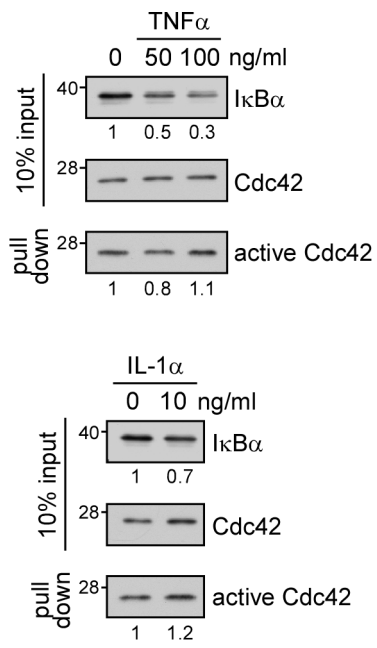

B

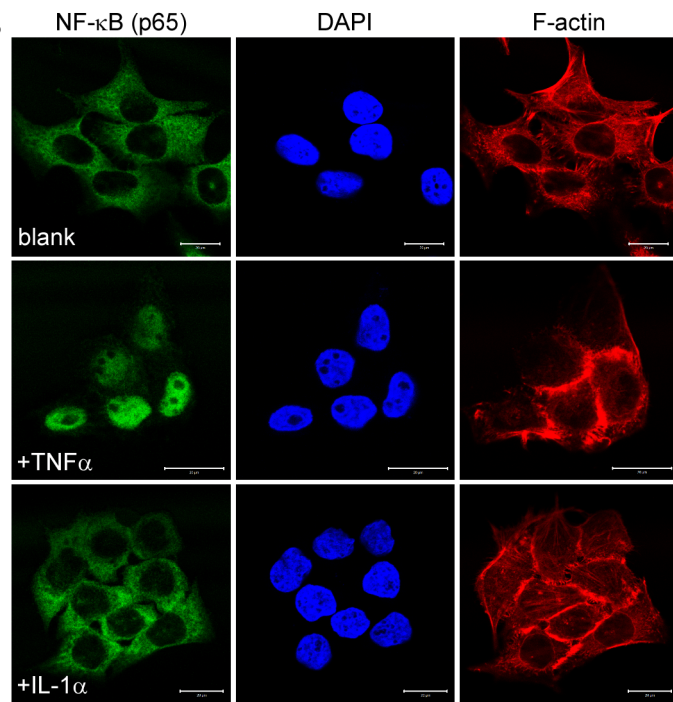

Supplement: Figure S1 — No apparent effect of TNF-α and IL-1α on Cdc42 activation in NPC cells. (A) Neither TNF-α nor IL-1α can induce Cdc42 activation. NPC-TW01 cells cultured for 24 h after seeding were treated with recombinant TNF-α (50 or 100 ng/ml) or with IL-1α (10 ng/ml) for 30 min under a serum-free condition. Cells without cytokine treatment were used as a control. Each cell lysate was then harvested for the GST-CBD pull-down assays to determine the level of active Cdc42. A reduced protein level of IκBα indicates the activation of NF-κB signaling under the indicated treatment. (B) No apparent effect of TNF-α and IL-1α on actin organization. NPC-TW04 cells that had been grown on poly-L-lysine-coated coverslips overnight were treated with TNF-α (100 ng/ml) or with IL-1α (10 ng/ml) for 30 min under a serum starvation condition. Then cells were fixed and permeabilized, followed by subsequent staining with primary anti-p65 and FITC-conjugated secondary antibodies. Cells were co-stained with TRITC-conjugated phalloidin to reveal the actin filaments. Nuclei were identified by DAPI staining. Translocation of p65 from the cytoplasm to the nuclei evidenced the activation of NF-κB signaling. Images were acquired using a ZEISS LSM510 confocal microscope. Scale bars, 20 µm. (PDF) [file ppat.1002690.s001.pdf]

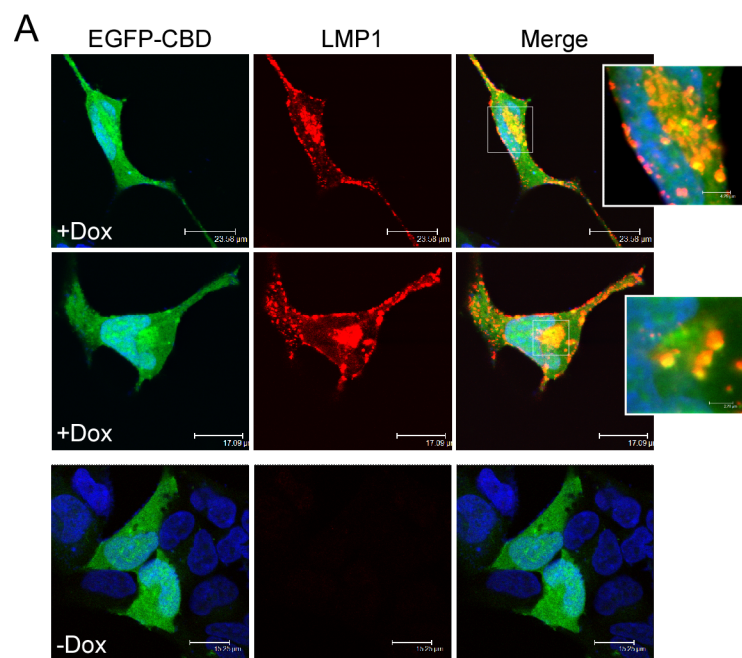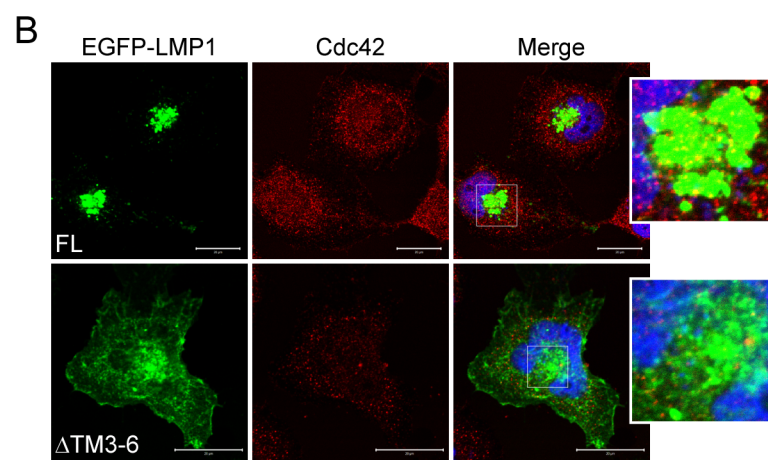

Supplement: Figure S2 — LMP1 appears to induce Cdc42 activation at LMP1-resident sites. (A) Spatial distribution of active Cdc42 upon LMP1 expression. 293 Tet-On cells that had been grown on poly-L-lysine-coated coverslips overnight were transfected with 1 µg of plasmid encoding an indicator for active Cdc42, EGFP-CBD, and incubated for 24 h with or without Dox (5 µg/ml) induction of LMP1 expression. Following a 6-h serum starvation, cells were fixed with 3.7% formaldehyde and subsequently stained with primary anti-LMP1 (S12) and TRITC-conjugated secondary antibodies. Nuclei were identified by DAPI staining (blue). Images were acquired using a ZEISS LSM510 confocal microscope. Scale bars, 15–17 µm. The insets were acquired at higher magnification (scale bars, 2–5 µm). (B) Co-localization of a portion of Cdc42 with LMP1. NPC-TW04 cells grown on coverslips overnight were transfected with 0.5 µg of plasmid for EGFP-LMP1 or its ΔTM3–6 truncated form and then incubated for 24 h. Following a 6-h serum starvation, cells were fixed and subsequently stained with a primary anti-Cdc42 antibody (P1, Santa Cruz; 1∶50 dilution) and a TRITC-conjugated secondary antibody. Nuclei were identified by DAPI staining (blue). Images were acquired using a ZEISS LSM510 confocal microscope. Scale bars, 20 µm. The insets demonstrated co-localization of a portion of Cdc42 with EGFP-LMP1 (yellow spots) rather than with the ΔTM3–6 form. (PDF) [file ppat.1002690.s002.pdf]

A

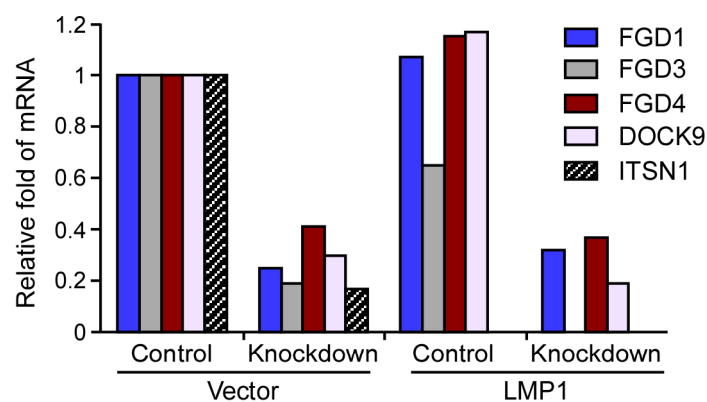

B

[illegible]

Supplement: Figure S3 — Knockdown of FGD4 in NPC cells and sequence comparison of FGD4 between species. (A) Knockdown efficiency of the targeted GEFs in NPC cells expressing LMP1 or transfected with empty vector (control). Total RNA isolated from cells of each treatment group was reverse transcribed and analyzed by quantitative RT-PCR using specific primer sets. The knockdown efficiency for each targeted GEF in each treatment is presented as a ratio of the mRNA level of each GEF in the knockdown cells divided by that in the respective control. (B) Sequence comparison of human, rat, and mouse FGD4. Shaded regions represent the conserved functional domains of FGD4. Asterisks (*) indicate identical amino-acid residues. (PDF) [file ppat.1002690.s003.pdf]

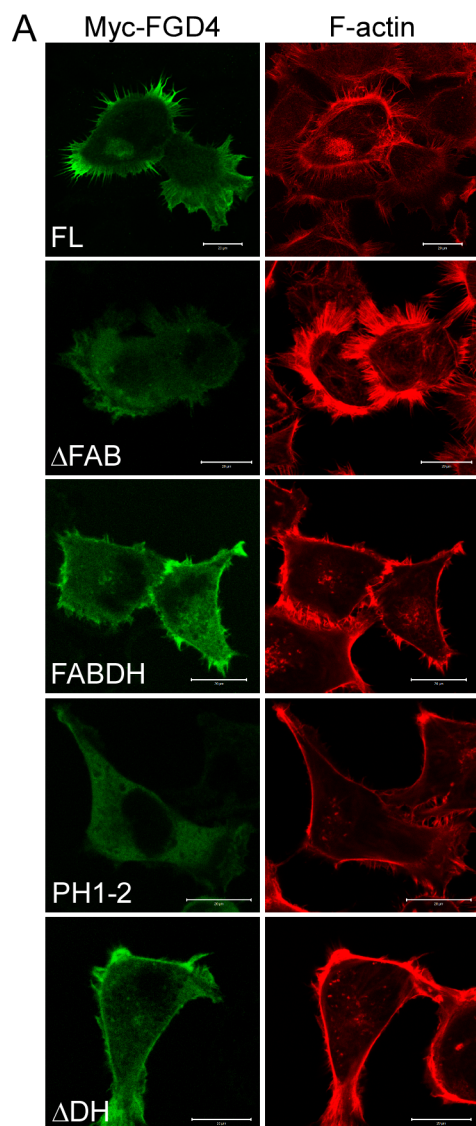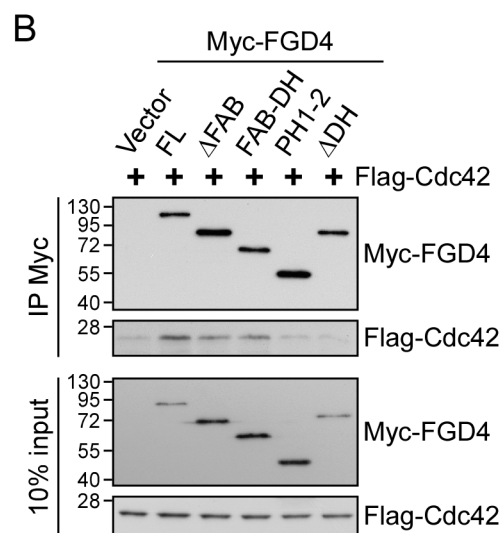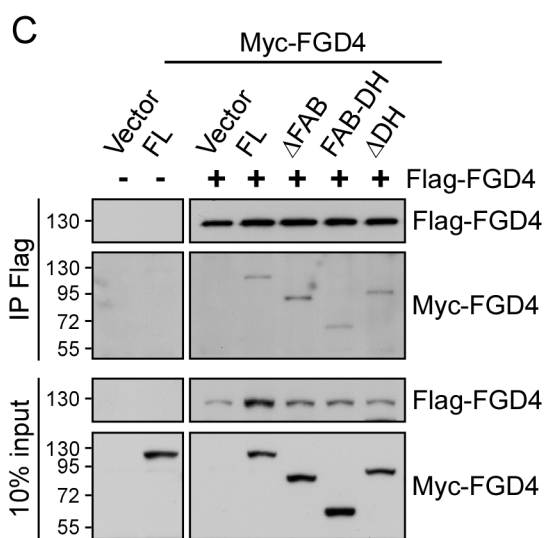

Supplement: Figure S4 — Functional characterization of FGD4. (A) NPC-TW01 cells grown on coverslips overnight were transfected with 0.5 µg of plasmid for Myc-FGD4 or its truncated forms and then incubated for 24 h. Following a 6-h serum starvation, cells were fixed and subsequently stained with a primary anti-Myc antibody (9E10) and a FITC-conjugated secondary antibody. Then the cells were co-stained with TRITC-conjugated phalloidin to indicate the actin filaments. Images were acquired using a ZEISS LSM510 confocal microscope. Scale bars, 20 µm. (B) FGD4 associates with Cdc42 mainly through the DH domain. NPC-TW01 cells were co-transfected with 1 µg of expression plasmid for Flag-tagged Cdc42 and 1 µg of expression plasmid for Myc-tagged full-length or truncated FGD4. At 24 h post-transfection, the cells were lysed and the resulting cell lysates were analyzed by co-immunoprecipitation using an anti-Flag affinity matrix. The precipitated proteins as well as unprecipitated lysates (input) were analyzed by Western blotting with anti-Flag and anti-Myc antibodies. (C) Self-association of FGD4. NPC-TW01 cells were co-transfected with 1 µg of expression plasmid for Flag-FGD4 and 1 µg of expression plasmid for Myc-FGD4 or its truncated forms. At 24 h post-transfection, the cells were lysed and the resulting cell lysates were subjected to co-immunoprecipitation assays using an anti-Flag affinity matrix, as described above. (PDF) [file ppat.1002690.s004.pdf]

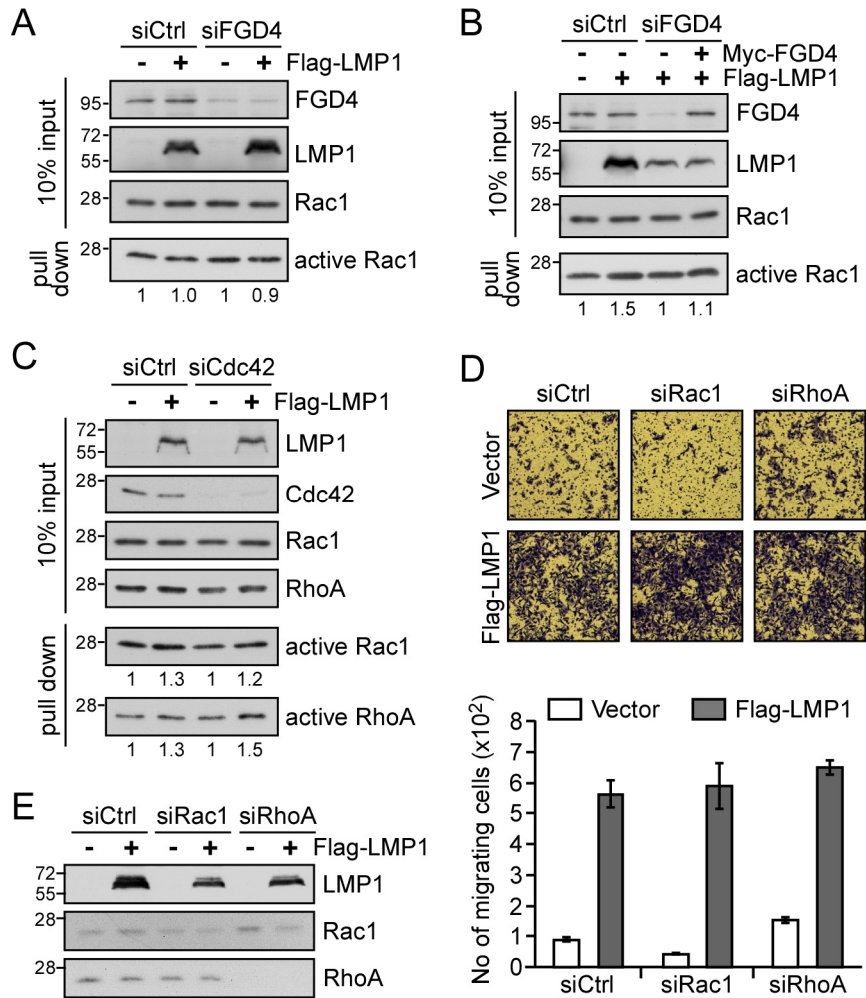

Supplement: Figure S5 — Rac1 and RhoA are not involved in the NPC cell motility mediated by the LMP1-FGD4-Cdc42 axis. (A) Knockdown or (B) re-introduction of FGD4 has no effect on Rac1 activation. NPC-TW04 cells were co-transfected with 25 µM control or FGD4 siRNA and 1 µg of expression plasmid for Flag-LMP1 or empty vector. In the case of re-introduction, 2 µg of Myc-FGD4 expression plasmids or Myc vector were added into the transfection. After 48-h incubation and a following 6-h serum starvation, cells were lysed for GST-PBD pull-down assays and analyzed for the level of active Rac1. (C) LMP1-induced Cdc42 activation is not associated with Rac1 or RhoA activation. NPC-TW02 cells were co-transfected with control or Cdc42 siRNA duplexes and 1 µg of expression plasmid for Flag-LMP1 or empty vector. After 48-h incubation and a following 6-h serum starvation, cells were lysed for GST-PBD and GST-RBD pull-down assays to analyze the levels of active Rac1 and active RhoA, respectively. (D) Rac1 and RhoA are not involved in LMP1-induced cell migration. NPC-TW02 cells were co-transfected with control, Rac1 or RhoA siRNA duplexes plus a plasmid for Flag-LMP1 or empty vector. Cells were then re-seeded for transwell migration assays as detailed above. (E) The knockdown efficiency was confirmed by Western blot analysis of a portion of cells used in transwell migration assays with anti-Rac1 and anti-RhoA antibodies. (PDF) [file ppat.1002690.s005.pdf]
