# Supplementary material for: Epstein-Barr Virus-Encoded LMP1 Interacts with FGD4 to Activate Cdc42 and Thereby Promote Migration of Nasopharyngeal Carcinoma Cells
Source: PLoS Pathog. 2012 May 10;8(5):e1002690. doi: 10.1371/journal.ppat.1002690 (PMC3349753; doi:10.1371/journal.ppat.1002690)
Supplement: Table S1 — Functional characterization of FGD4 domains and their respective effects on LMP1-mediated Cdc42 activation. (PDF) [file ppat.1002690.s006.pdf]

Table S1. Functional characterization of FGD4 domains and their respective effects on LMP1-mediated Cdc42 activation

	FGD4 constructs				
	Full length	ΔFAB	FAB-DH	PH1-2	ΔDH
Activation of Cdc42 *in vivo*[a]	Yes	Yes	Impaired	Impaired	Impaired
Enhanced function of LMP1[b]	Yes	Yes	Impaired	Impaired	Impaired
Association with LMP1[c]	Yes	Yes	Impaired	Yes	Yes
Association with Cdc42[b]	Yes	Yes	Yes	Impaired	Impaired
Self-association with FGD4[e]	Yes	Yes	Yes	ND[f]	Yes

[a] Activation of Cdc42 in NPC cells determined by GST-CBD-pulldown assays.
[b] LMP1-mediated Cdc42 activation was elevated by co-expression with individual FGD4 construct in NPC cells.
[c] Demonstrated by co-immunoprecipitation assays in NPC cells or *in vitro* GST-FGD4- pulldown assays.
[d] Demonstrated by co-immunoprecipitation assays in NPC cells.
[e] Demonstrated by co-immunoprecipitation assays in NPC cells.
[f] Not determined.
