# Supplementary material for: Epstein-Barr Virus-Encoded LMP1 Interacts with FGD4 to Activate Cdc42 and Thereby Promote Migration of Nasopharyngeal Carcinoma Cells
Source: PLoS Pathog. 2012 May 10;8(5):e1002690. doi: 10.1371/journal.ppat.1002690 (PMC3349753; doi:10.1371/journal.ppat.1002690)
Supplement: Table S2 — Clinical information for the nasopharyngeal biopsies used in the quantitative RT-PCR and immunohistochemistry analyses. (PDF) [file ppat.1002690.s007.pdf]

Table S2. Clinical information for the nasopharyngeal biopsies used in the quantitative RT-PCR and immunohistochemistry analyses

Patient	Gender	Age (years)	TNM[a]	Stage[b]	WHO[a] type[c]
NPC026	M	43	T2bN2M0	III	III
NPC027	M	42	T1N0M0	I	II
NPC031	M	61	T3N1M0	III	III
NPC033	M	66	T1N1M0	IIb	III
NPC034	M	41	T2bN2M0	III	III
NPC035	M	80	T3N0M0	III	III
NPC036	M	49	T4N2M0	IVa	III
NPC037	M	69	T2bN0M0	IIb	III
NPC040	M	43	T1N1M0	IIb	III
NPC041	M	47	T4N0M0	IVa	III
NPC043	M	63	T1N1M0	IIb	III
NPC044	M	42	T2N2M0	III	III
NPC047	M	25	T2N2M0	IIb	II
NPC016	M	33		IVa	III
NPC020	M	40		IIb	II
NPC022	M	45		IVb	II
NPC028	M	65		III	III

[a] Abbreviations: TNM, tumor-node-metastasis; WHO, world health organization.
[b] According to American Joint Committee on Cancer 1997 staging system.
[c] WHO type II, nonkeratinizing squamous cell carcinoma; WHO type III, undifferentiated carcinoma.
