# Supplementary material for: Epstein-Barr Virus-Encoded LMP1 Interacts with FGD4 to Activate Cdc42 and Thereby Promote Migration of Nasopharyngeal Carcinoma Cells
Source: PLoS Pathog. 2012 May 10;8(5):e1002690. doi: 10.1371/journal.ppat.1002690 (PMC3349753; doi:10.1371/journal.ppat.1002690)
Supplement: Table S3 — Primer sets for generating expression vectors. (PDF) [file ppat.1002690.s008.pdf]

Table S3. Primer sets for generating expression vectors

Construct	Amino acids (aa)	Sense (5' to 3')	Antisense (3' to 5')
hFGD4	1-766 (full length)	ATGGAGGAAATTAAACCTGC	TCAGCATTCTGATTTTTTCTTAGG
ΔFAB	151-766	GCTTCTGACAGTAGCTACAGGAC	TCAGCATTCTGATTTTTTCTTAGG
FAB-DH	1-401	ATGGAGGAAATTAAACCTGC	TCACTTTAGGTTCTCCATTTTCCT
PH1-2	401-766	AAGAAACTCTTAGAGATTTATGAAATG	TCAGCATTCTGATTTTTTCTTAGG
ΔDH	Δ201-400	GCACCATGAGATGAAGAAGAAACTCTTAGAGA	TCTCTAAGAGTTTCTTCTTCATCTCATGGTGC
FAB-PH1	1-539	ATGGAGGAAATTAAACCTGC	TCAGTCATTATCCTTTGCAATTGCA
CBD(WASP)	201-321	CTCAAGCTTGACATCCACAACCCTGAC	CCGGGATCCTCGAGATGGCGGTGGGGG
Cdc42	full length	CGGAATTCAATGCAGACAATTAAGTGTGTTG	CGGGATCCTCATAGCAGCACACACCTG
