# Supplementary material for: Epstein-Barr Virus-Encoded LMP1 Interacts with FGD4 to Activate Cdc42 and Thereby Promote Migration of Nasopharyngeal Carcinoma Cells
Source: PLoS Pathog. 2012 May 10;8(5):e1002690. doi: 10.1371/journal.ppat.1002690 (PMC3349753; doi:10.1371/journal.ppat.1002690)
Supplement: Table S4 — Primer sets for quantitative RT-PCR. (PDF) [file ppat.1002690.s009.pdf]

Table S4. Primer sets for quantitative RT-PCR

Protein name	Gene symbol	Sense (5' to 3')	Antisense (5' to 3')
LMP1	*LMP1*	ACAAAACTGGTGGACTC	GTCTGCCCTCGTTGGA
DOCK9	*DOCK9*	CCTGAGACGACAGGGTCGATA	TGCCAGTCAGAGTTATAGGTTTTGA
Intersectin-1	*ITSN1*	GGCATTGGAAGGTTGATGGT	AACCCATGGTCACCTCACAGTAC
FGD4 (Frabin)	*FGD4*	GCCAATGAACTTTTGCTTACTGAA	CGAGCCTCGGTTTGCTTCT
FGD1	*FGD1*	ATCCCCCGCTATGAGCTTCT	TGGCGATCAGCTCCAGAGA
FGD3	*FGD3*	ACAGAGGAAGAGAAGAAAGAATGGAT	TGAAGGCGCCACCAAAAG
Collagen, type IV, alpha	*COL4A6*	AGAGGTCAGCACACAT	GCTTTACTTTGAACCAGGC
GADPH	*GADPH*	GAAGGTGAAGGTCGGAGTC	GAAGATGGTGATGGGATTTC
